# A clinical KPC-producing *Klebsiella michiganensis* strain carrying IncFII/IncFIA (HI1)/IncFIB (K) multiple replicon plasmid

**DOI:** 10.3389/fmicb.2022.1086296

**Published:** 2023-01-04

**Authors:** Na Zhang, Xiong Liu, Lihua Qi, Jiali Chen, Shiyu Qin, Meiling Jin, Xiaojing Yang, Fangni Liu, Jinpeng Guo, Jie Liu, Changjun Wang, Yong Chen

**Affiliations:** ^1^School of Public Health, China Medical University, Shenyang, Liaoning province, China; ^2^Department of Emergency Response, Chinese PLA Center for Disease Control and Prevention, Beijing, China; ^3^Department of Information, Chinese PLA Center for Disease Control and Prevention, Beijing, China; ^4^Department of Clinical Laboratory, Seventh Medical Center of Chinese PLA General Hospital, Beijing, China; ^5^College of Public Health, Zhengzhou University, Zhengzhou, Henan province, China

**Keywords:** *Klebsiella michiganensis*, genome analysis, evolution, plasmid, carbapenemase

## Abstract

*Klebsiella michiganensis* is an increasingly important bacterial pathogen causing nosocomial infections in clinical patients. In this study, we described the molecular and genomic characteristics of a carbapenem-resistant *K. michiganensis* strain KM166 cultured from a one-month premature baby’s blood sample. KM166 showed lower biofilm forming ability in optical density (OD) than *K. pneumoniae* NTUH-K2044 (0.271 ± 0.027 vs. 0.595 ± 0.054, *p* = 0.001), and the median lethal dose (0.684 lg CFU/mL) was lower than *K. pneumoniae* strain NTUH-K2044 (6.679 lg CFU/mL). A IncFII/IncFIA(HI1)/IncFIB(K) multiple replicon plasmid in KM166 was identified carrying three replicon types. It has low homology to *Escherichia coli* pMRY09-581ECO_1 and the highest homology similarity to the INcFIA/INcFII(p14)-type plasmid in *K. michiganensis* strain fxq plasmid pB_KPC, suggesting that this multiple replicon plasmid was unlikely to have been transmitted from *E. coli* and probably a transfer of *repFIB* replicon genes from other *K. michiganensis* strains into the INcFIA/INcFII(p14)-type plasmid of KM166 had occurred. Mapping of the gene environment revealed that *bla*_KPC-2_ in KM166 plasmid 3 had high identity and same Tn3-tnpR-IS481-*bla*_KPC-2_-klcA_1 genomic context structure with *K. pneumoniae* strain JKP55, plasmid pKPC-J5501, and *bla*_KPC-2_-carrying plasmid proved to be autonomously transferred under the help of mobile genetic elements into *Escherichia coli* 600 by plasmid conjugation experiment. In conclusion, we have characterized a *K. michiganensis* strain carrying multi-replicon IncFII/IncFIA(HI1)/IncFIB(K) plasmid and *bla*_KPC-2_-carrying IncFII(p14)/IncFIA plasmid in this study, which provided insights about the evolutionary diversity of plasmids carried by *K. michiganensis*.

## 1. Introduction

*Klebsiella michiganensis* is one of the bacterial pathogen of the *Klebsiella* genus (Wyres et al., 2020), it seldom appeared in clinical hospitals at the past, but has been gradually increasing in recent years, for several *K. michiganensis* strains have been found to be colonized in patients. Carbapenemase production is the common carbapenemase resistance mechanism in clinical infections, such as *Acinetobacter baumannii* (Oinuma et al., 2021), *K. pneumoniae* (Pitout et al., 2015) and *Pseudomonas aeruginosa* (Nordmann et al., 2011; Doi and Paterson, 2015; Iovleva and Doi, 2017). The main carbapenemase genes carried by *K. michiganensis* include *bla*_KPC-2_, *bla*_VIM-1_ (Campos-Madueno et al., 2021), *bla*_NDM-1_ (Li et al., 2021)，*bla*_NDM-5_ (Zheng et al., 2018), *bla*_KPC-3_ (Seiffert et al., 2019), *bla*_IMP-4_ (Li et al., 2021; Zhang et al., 2021), *bla*_OXA-181_ (Founou et al., 2018)，*bla*_SIM-1_, *bla*_OXA-1_, *bla*_CTX-M-14_ (Xu et al., 2022), etc.

*Klebsiella michiganensis* has appeared in parts of the world (Ccorahua-Santo et al., 2017; King et al., 2021; Luo et al., 2022), there are highly homology and similarities in the molecular characteristics between *K. michiganensis* and *K. pneumoniae*. Some *K. michiganensis* strains have been found in plants or ecosystems (Kang et al., 2020; Salgueiro et al., 2020; Liu, L. et al., 2021), but the majority have been found in patients (Abed et al., 2021). Due to the cadmium resistance and plant growth promoting properties of *K. michiganensis*, many studies have been conducted to examine the effects of physical and biochemical stimuli such as PH, cadmium and environmental changes on the growth and genome sequence of *K. michiganensis*, with many positive results obtained (Mitra et al., 2018, 2019; Oliveira et al., 2020; Yan et al., 2021). However, more research is needed to examine the genomic characteristics of *K. michiganensis* strains from clinical patients, as well as its drug resistance and virulence profile (Zhang et al., 2021).

## 2. Materials and methods

### 2.1. Sample sources and strain identification

A total of 359 strains isolated from a tertiary hospital in Beijing, China were identified to be *K. pneumoniae* through biochemical indicators during 2017–2020, and 147 strains were resistant to carbapenem antibiotics. One of these 147 carbapenem resistant strain (KM166) was identified as *K. michiganensis* after whole genome sequencing. KM166 strain was cultured from a one-month premature baby’s blood on July 31, 2018, with a utilization history of penicillin, monocyclic β-lactams and cephalosporins. The patient was admitted on June 23, 2018 and discharged on September 5, 2018, with a hospitalization period of 75 days and had been treated with central venous cannulation, ventilator and blood transfusion. KM166 was cultured in a Luria-Bertani (LB) solid medium for 18–24 h in a constant temperature incubator at 37°C, the single colony was picked into LB liquid medium and incubated in a constant temperature shaker at 37°C for 18 h before being washed.

### 2.2. Antibiotic sensitivity and hypermucoviscous test

The sensitivity of common clinical antibiotics was tested using the Vitek-2 compact system and agar dilution method. KM166 strain were incubated on Columbia blood agar medium (Oxoid, Basingstoke, England) at 37°C for 18–24 h. Control strains *Pseudomonas aeruginosa* ATCC 27853 and *Escherichia coli* ATCC 25922 were used for antimicrobial susceptibility testing. The MICs were verified according to the Clinical and Laboratory Standards Institute (M100, 29th edition, 2019) guidelines. Standard bacteriological rings were used to assess hypermucoviscous phenotype, and the formation of slimy lines >5 mm in length was considered a positive string test (Catalán-Nájera et al., 2017).

### 2.3. Biofilm formation capacity test

Biofilm formation was measured by the absorbance at 570 nm of crystal violet staining. Hypervirulence *K. pneumoniae* strain NTUH-K2044 was served as the control and negative control was the group without the addition of bacterial solution. The strain was incubated in LB liquid medium until it reached a logarithmic growth cycle and then washed twice, followed by adjustment of the bacterial liquid to 1.0 McFarland’s concentration solution, then 200 μl LB culture solution and 10 μl bacterial solution were added to each well of the 96-well cell culture plate, and the 96-well cell culture plate was placed in a wet box at 35°C for 48 h. Afterwards, 200 μl of 1% crystalline violet staining solution was added to each well waiting for 10 min, and the plate was washed twice with distilled water and dried. The solution was transferred to a new 96-well plate and the absorbance value at 570 nm (A570) was observed using an enzyme marker (Liu, Y. et al., 2019).

### 2.4. The neutrophil killing assay

Serum from healthy people (#9193-10ML, purchased from Lablead, Beijing) was collected. The strain was cultured on Columbia blood agar medium (Oxoid, Basingstoke, England) for 15–18 h overnight and cultured in LB liquid medium for 18 h, washed twice with PBS buffer and then diluted to 1 × 10^6^ CFU/ml. Mixtures of 75 μl serum and 25 μl diluted bacterial solution were collected at 37°C on a constant temperature shaker for 0 h, 1 h, 2 h, 3 h, and 4 h (Siu et al., 2011). The classical *K. pneumoniae* strain NTUH-K2044 was used as a control in the experiment, and observations were made to assess the bacterial killing effect of the serum by counting the average number of viable bacteria after taking 10 μl bacterial solution and applying it to three LB agar medium at different observation times.

### 2.5. The *Galleria mellonela* model

The larvae of *Galleria mellonella* (purchased from Tianjin Huiyude Biotechnology Co., Ltd.) that met the test standards were purchased, each weighing about 300–400 mg. There were two control groups: one received the same amount of PBS and the other was not treated. The strains were grown on LB agar medium for 18–20 h before the bacterial solution was diluted to 10^4^–10^7^ CFU/ml with PBS. A group of 10 *G. mellonella* was chosen at random, each *K. michiganensis* strain was injected into three groups and four different dilution concentrations (totally 120 *G. mellonella*). The hypervirulence *K. pneumoniae* strain NTUH-K2044 served as the control. A total of 20.0 μl bacterial suspension was injected into the first abdominal segment of *G. mellonella* using microinjector, then the death number of each group was observed and recorded every 6 h until 72 h. The median lethal dose (1og10 CFU/mL) of strains was calculated using the probit probability method of regression analysis (Gu et al., 2018).

### 2.6. Plasmid conjugation experiments and strain identification

KM166 carrying *bla*_KPC-2_ as donor and *E. coli* EC600 as recipient (Cai et al., 2008) were co-inoculated on dual resistant BHI solid medium (Meropenem 4 ug/mL and Rifampicin 2 mg/mL) and incubated for 48 h at 37°C. Colonies were grown and amplified using *bla*_KPC-2_ primers (F: CGGGTTGGACTCAAGACG; R: TGATGCGGTATTTTCTCC) to determine the *bla*_KPC-2_ in the conjugates. The conjugation bacterial species were subsequently identified using Vitek-2 compact system.

### 2.7. Genomic DNA extraction, sequencing and assembly

Strain DNA was extracted using four steps for long fragment DNA (lysis, purification, precipitation and elution). Pacbio sequel II and the DNBSEQ platform were used to sequence the genome of KM166. An insert DNA of 350 bp was identified to perform genome sequencing and the original PacBio long reads were error-corrected, trimmed and assembled using the program Canu v1.5 (Koren et al., 2017). The raw data was treated in order to obtain more accurate and reliable results in subsequent bioinformatics analysis: (1) remove reads containing a certain proportion of low quality (≤20) bases (40% as default); (2) removal of reads with a total number of bases containing N up to 10%; (3) remove adapter contamination; (4) remove duplication contamination.

### 2.8. Genome component prediction, gene annotation and protein classification

Species identification was performed using Kleborate software (Lam et al., 2021) to obtain multilocus sequence types (MLST). Genome annotation was performed using prokka v1.14.6 (Seemann, 2014). tRNA, rRNA and sRNA recognition were made use of tRNAscan-SE (Chan et al., 2021), Barrnap and the Rfam database. Prophage regions were predicted using the PHAgeSearch Tool (PHAST) web server^1^ and CRISPR identification using CRISPRCasFinder.^2^ Virulence and resistance genes were identified based on the database in VFDB (Virulence Factor Database; Liu, B. et al., 2019) and CARD (The Comprehensive Antibiotic Research Database; Alcock et al., 2020) using ABRicate.

### 2.9. Comparative genomics and phylogenetic analysis

The *K. michiganensis* strain K.m.2654 (accession no. GCA_003011775.1) isolated from Denmark in 2017 was selected for comparative genomic analysis with KM166. The comparison of K.m.2654 and KM166 was performed using blast and the average nucleotide identity comparisons between genomes were calculated using FastANI. The phylogenetic tree was constructed by maximum likelihood method based on single nucleotide polymorphism (SNP) using ClonalFrameML v1.12 (Didelot and Wilson, 2015) and roary v3.13.0. The tree building model was calculated by ModelTest-NG v0.1.7 (Flouri et al., 2015; Darriba et al., 2020) and plotted by RaxML-NG v1.1.0 (Kozlov et al., 2019), and the phylogenetic tree was finally visualized by FigTree v1.4.3 and iTOL (Letunic and Bork, 2021).

## 3. Results

### 3.1. Antimicrobial resistance and virulence phenotype

**Figure 1:**
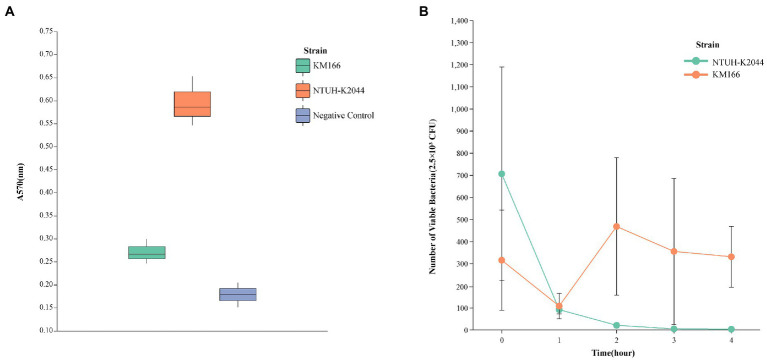
Defensive capability test of *Klebsiella michiganensis* KM166. **(A)** biofilm formation capacity test. **(B)** killing experiment with human neutrophils.

The *in vitro* antimicrobial susceptibility testing showed that *K. michiganensis* KM166 was resistant to Ampicillin, Aztreonam, carbapenems, and so on (Table 1). The string test showed negative result, which suggested that KM166 was not a hypermucoviscous strain. Both KM166 and classical *K. pneumoniae* strain NTUH-K2044 showed positive biofilm-forming ability (Figure 1A), but KM166 showed lower biofilm forming ability in optical density (OD) than *K. pneumoniae* NTUH-K2044 (0.271 ± 0.027 Vs. 0.595 ± 0.054, *p* = 0.001). The results of the serum killing experiment showed that human serum had a weak killing capacity against KM166, and KM166 has the stronger serum bactericidal capacity after 1 hour of serum and bacterial solution were mixed (Figure 1B). The median lethal dose of KM166 was 0.684 lg CFU/mL, which was much lower than the *K. pneumoniae* NTUH-K2044 strain (median lethal dose: 6.679 lg CFU/mL).

**Table 1:** Resistance of *Klebsiella michiganensis* KM166 to commonly used clinical antibiotics.

Antibiotics	MIC (μg/mL)	Resistance results (R, I, S)	Antibiotics	MIC (μg/mL)	Resistance results (R, I, S)
Penicillins			Carbapenems		
Ampicillin	≥32	R	Ertapenem	>16	R
Artificial synthetic antibiotics			Imipenem	>8	R
Ampicillin/Sulbactam	≥32	R	Meropenem	>4	R
Piperacillin/tazobactam	16	S	Aminoglycosides		
Cephalosporins			Gentamycin	≤1	S
Ceftazidime	4	S	Amikacin	≤2	S
Ceftriaxone	≥64	R	Tobramycin	≤1	S
Cefepime	≥64	R	Quinolones		
Monocyclic β-lactams			Ciprofloxacin	1	S
Aztreonam	16	R	Levofloxacin	1	S

R, resistance; I, intermediate; S, susceptible.

### 3.2. Genomic characterization

A total of 1,297 Mb whole-genome sequencing reads (187X) and 5,159 Mb (745X) PacBio platform reads were generated. The total genome of *K. michiganensis* KM166 has a size of 6,917,102 bp and contains 6,765 genes, with a GC content of 55.19 percent. The complete genome consists of a 6,164,986-bp single chromosome and four plasmids including Plasmid 1(359,068 bp), Plasmid 2(204,198 bp), Plasmid 3(141,565 bp) and Plasmid 4(47,285 bp). The G + C contents of the chromosome and the four plasmids were 55.78, 48.32, 51.96, 53.61 and 49.36%, respectively (Table 2). The plasmid types identified in KM166 were TrfA, IncFII/IncFIA(HI1)/IncFIB(K), IncFII(p14)/IncFIA and IncN3.

**Table 2:** Genome assembly information of *Klebsiella michiganensis* KM166.

ID name	Sequence topology	Total length (bp)	GC content (%)	Plasmid type
Chromosome 1	circular	6,164,986	55.78	/
Plasmid 1	circular	359,068	48.32	TrfA
Plasmid 2	circular	204,198	51.96	IncFII/IncFIA(HI1)/IncFIB(K)
Plasmid 3	circular	141,565	53.61	IncFII(p14)/IncFIA
Plasmid 4	circular	47,285	49.36	IncN3

A total of 168 ncRNA (85 tRNAs, 25 rRNAs and 58 sRNAs) and 290 repeats were identified. In addition, three CRISPRs were identified in the chromosome of KM166, with lengths of 90 bp, 97 bp and 111 bp, respectively. Eleven prophages were identified on chromosomes and one on plasmid 3. It was found that the serotype of KM166 was KL41, three resistance genes *bla*_OXY-1_, *bla*_CTX-M-37_ and *bla*_KPC-2_ were present in chromosome, IncN3 plasmid and IncFII(p14)/IncFIA plasmid, respectively.

### 3.3. Comparison of the nucleic acid identity

**Figure 2:**
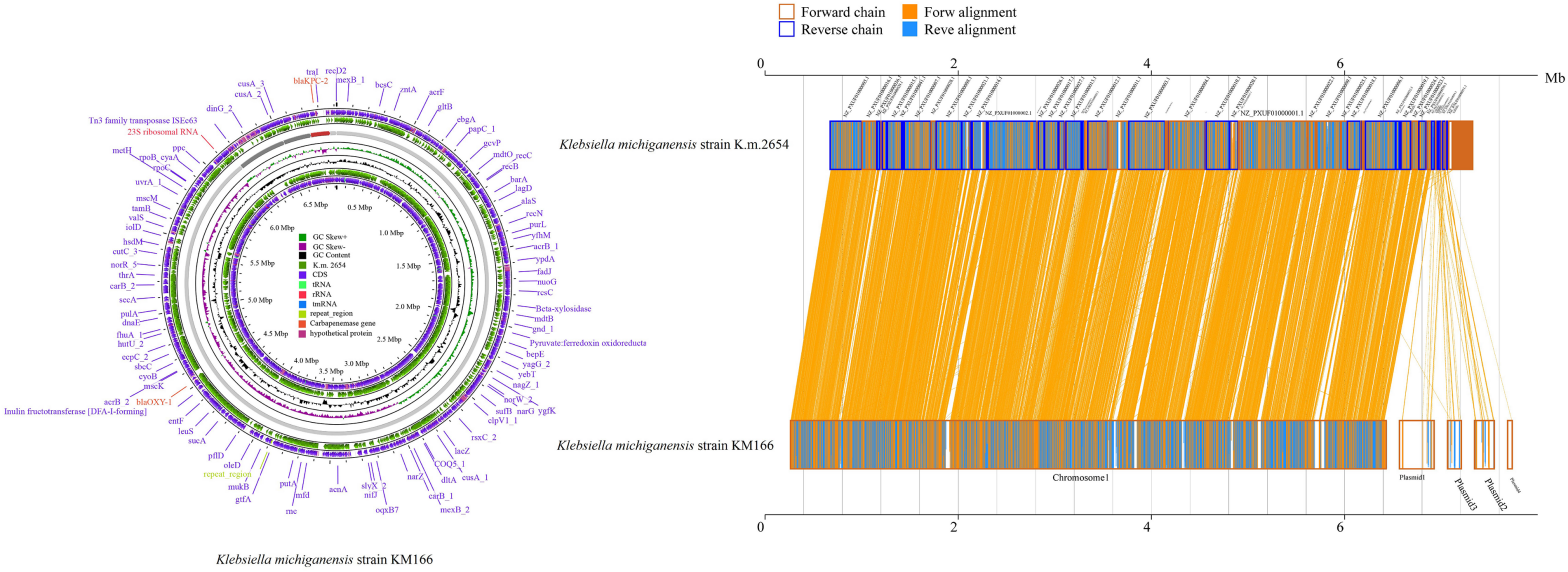
Amino acid level synteny analysis of *Klebsiella michiganensis* K.m.2654 and *K. michiganensis* KM166. Comparative genomic circle map was created using CGview (https://proksee.ca/). The yellow line in the middle region of the two sequences in the amino acid level synteny map indicates the forward alignment, and the blue line indicates the reverse complementary alignment.

Two clinical *K. pneumoniae* strains BSIKPN-9 and BSIKPN-11 collected from the same hospital as KM166 were randomly selected for comparison, and the average nucleotide identity (ANI) for *K. michiganensis* KM166 and *K. pneumoniae* (BSIKPN-9 and BSIKPN-11) were 84.64 and 84.65%, the range of which was between 83 and 95% of interspecific relationships. The ANI for KM166 and *K. michiganensis* strain K.m.2654 (accession no.GCF_003011775.1), on the other hand, was 99.81%, indicating intraspecific variation. Amino acid level synteny analysis of K.m.2654 and KM166 revealed that KM166 had 6,765 genes and K.m.2654 had 5,531 genes, with 5,052 aligned genes (74.68%, 5052/6765). Chromosomes of K.m.2654 and KM166 were found to be very similar, and the variants were mostly found in four plasmids (Figure 2).

### 3.4. Global evolutionary analysis of Klebsiella michiganensis

**Figure 3:**
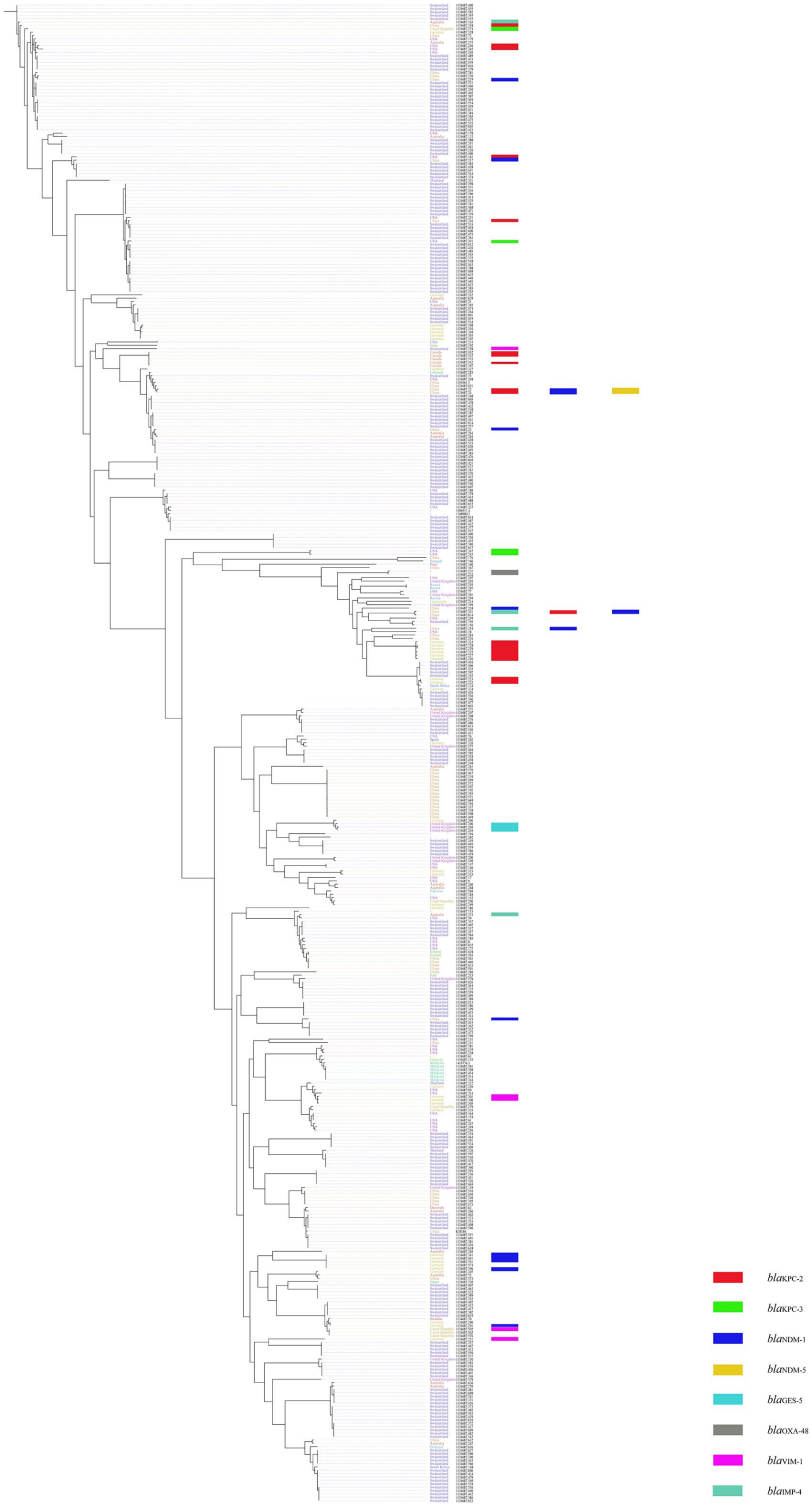
Phylogenetic tree of 445 *Klebsiella michiganensis* strains downloaded from Pathosystems Resource Integration Center and one *Klebsiella michiganensis* strain KM166 we collected. Plotted using ClonalFrameML and roray based on SNP. “-” means the collection of strains from unknown cities.

A total of 446 complete genomic assemblies classified as *K. michiganensis* were used to construct the phylogenetic tree, of which 445 downloaded from the Pathosystems Resource Integration Center (PATRIC), as well as one KM166 we collected in this study. Genomic information of 445 strains is provided in Supplementary Table S1. All 446 *K. michiganensis* strains were isolated from 27 countries, Switzerland was the main country source (52.24%, 233/446), followed by China with 50 strains (11.21%, 50/446). Five *K. michiganensis* strains were isolated from Beijing, China, but they did not carry *bla*_KPC-2_. The phylogenetic tree revealed a single major group, and the homology between strains was extremely high, with no significant evolutionary variation. KM166 was found at the end of an evolutionary branch that included some of the most recent strains, with the highest homology to a *K. michiganensis* strain collected in Switzerland (ANI: 99.93%). KM166 was collected from a hospital in Beijing on July 31, 2018, and several *K. michiganensis* strains with the highest homology were collected in 2017 (Figure 3).

### 3.5. Comparative analysis of the *bla*_KPC-2_-harbouring *Klebsiella michiganensis*

**Figure 4:**
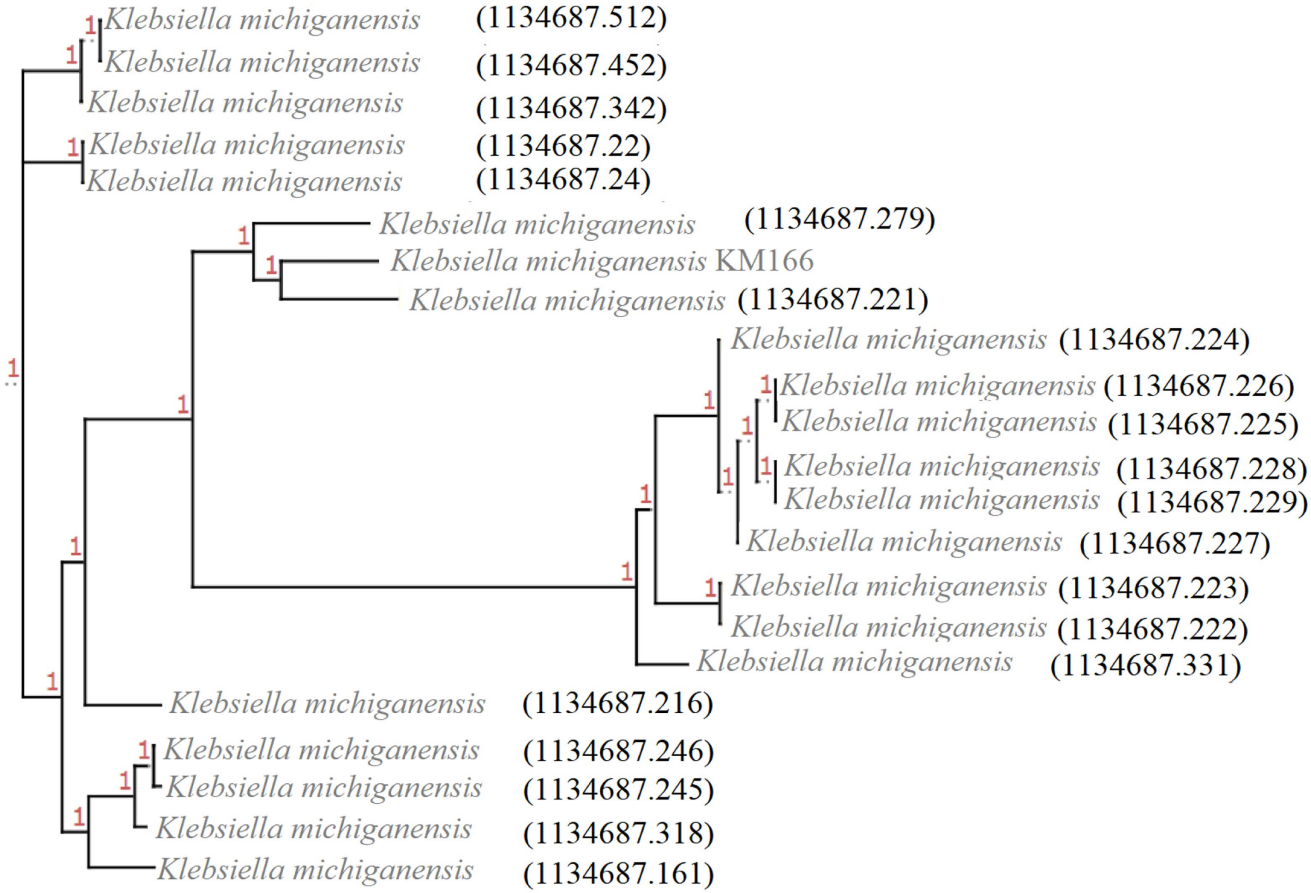
Phylogenetic tree of 22 *Klebsiella michiganensis* strains carrying *bla*_KPC-2_. The phylogenetic tree was generated using ClonalFrameML and roray based on SNP, plotted by treeview (http://etetoolkit.org/treeview/). 21 *Klebsiella michiganensis* strains carrying *bla*_KPC-2_ was identified from 445 *K. michiganensis* strains downloaded from Pathosystems Resource Integration Center and one *K. michiganensis* strain KM166 carrying *bla*_KPC-2_ was collected in this study.

Twenty-one *bla*_KPC-2_-carrying *K. michiganensis* strains were identified from 445 *K. michiganensis* strains downloaded from the PATRIC database, and they were then combined with KM166 for phylogenetic analysis in order to investigate their evolutionary relationships (Figure 4). It showed that two strains (1134687.279 and 1134687.221) isolated from the Czech Republic in 2018 and Germany in 2017 that shared high homology with KM166, particularly 1134687.221 (ANI: 99.15%). According to the phylogenetic tree, KM166 was located at a small branch and showed highly homologous to two strains isolated from geographically distant countries.

**Figure 5:**
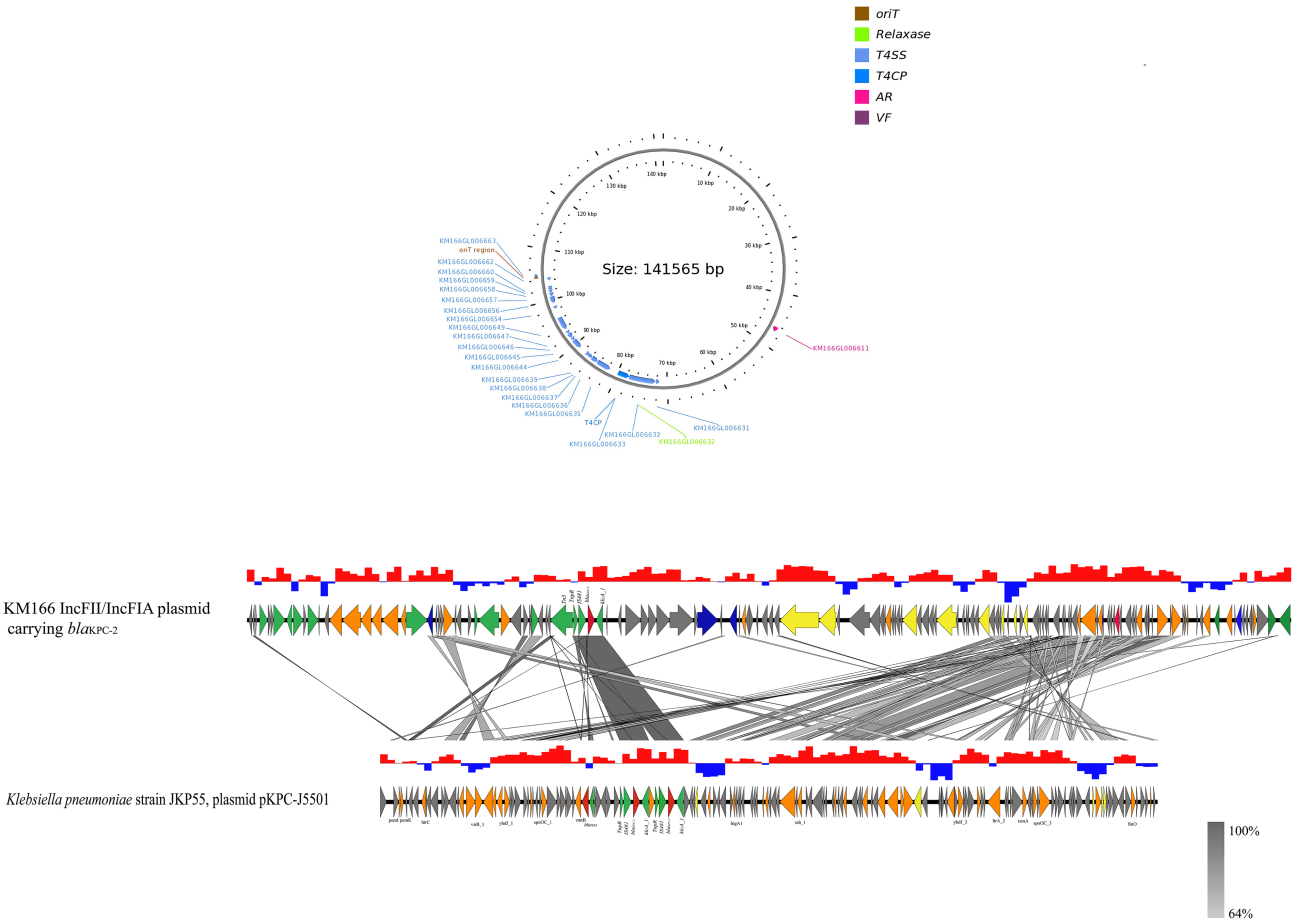
Sequencing comparison among the regions surrounding *bla*_KPC-2_ from KM166 IncFII(p14)/IncFIA plasmid and *Klebsiella pneumoniae* strain JKP55, plasmid pKPC- J5501, the most closely genetically matched complete plasmid sequences from NCBI. High homology shared sections are shaded in gray. An arrow is colored in accordance with its ostensible function. The function of genes about coupling transfer, plasmid stability, replication, mobile elements, antimicrobial resistance are yellow, orange, blue, green and red arrows, respectively. Genes that encode putative proteins and proteins with unknown functions are indicated by grey arrows.

The *bla*_KPC-2_ gene of KM166 was located in a 140 kb IncFII(p14)/IncFIA type plasmid 3, blasting with the nucleic acid database (NT) found that a plasmid named *K. pneumoniae* strain JKP55, plasmid pKPC-J5501(GenBank accession no. NZ_OL891656) showed 17% coverage and 99.97% identity to KM166 plasmid 3. Mapping of the gene environment using easyfig v2.2.5 (Sullivan et al., 2011) software revealed that *bla*_KPC-2_ in KM166 plasmid 3 had high identity and same Tn3-tnpR-IS481-*bla*_KPC-2_-klcA_1 genomic context structure with *K. pneumoniae* strain JKP55, plasmid pKPC-J5501, which carried two *bla*_KPC-2_ genes (Figure 5). Plasmid conjugation experiment demonstrated that the plasmid carrying *bla*_KPC-2_ could transfer autonomously into *Escherichia coli* 600.

### 3.6. Gene environment characteristics of IncFII/IncFIA(HI1)/IncFIB(K) multiple replicon plasmid

**Figure 6:**
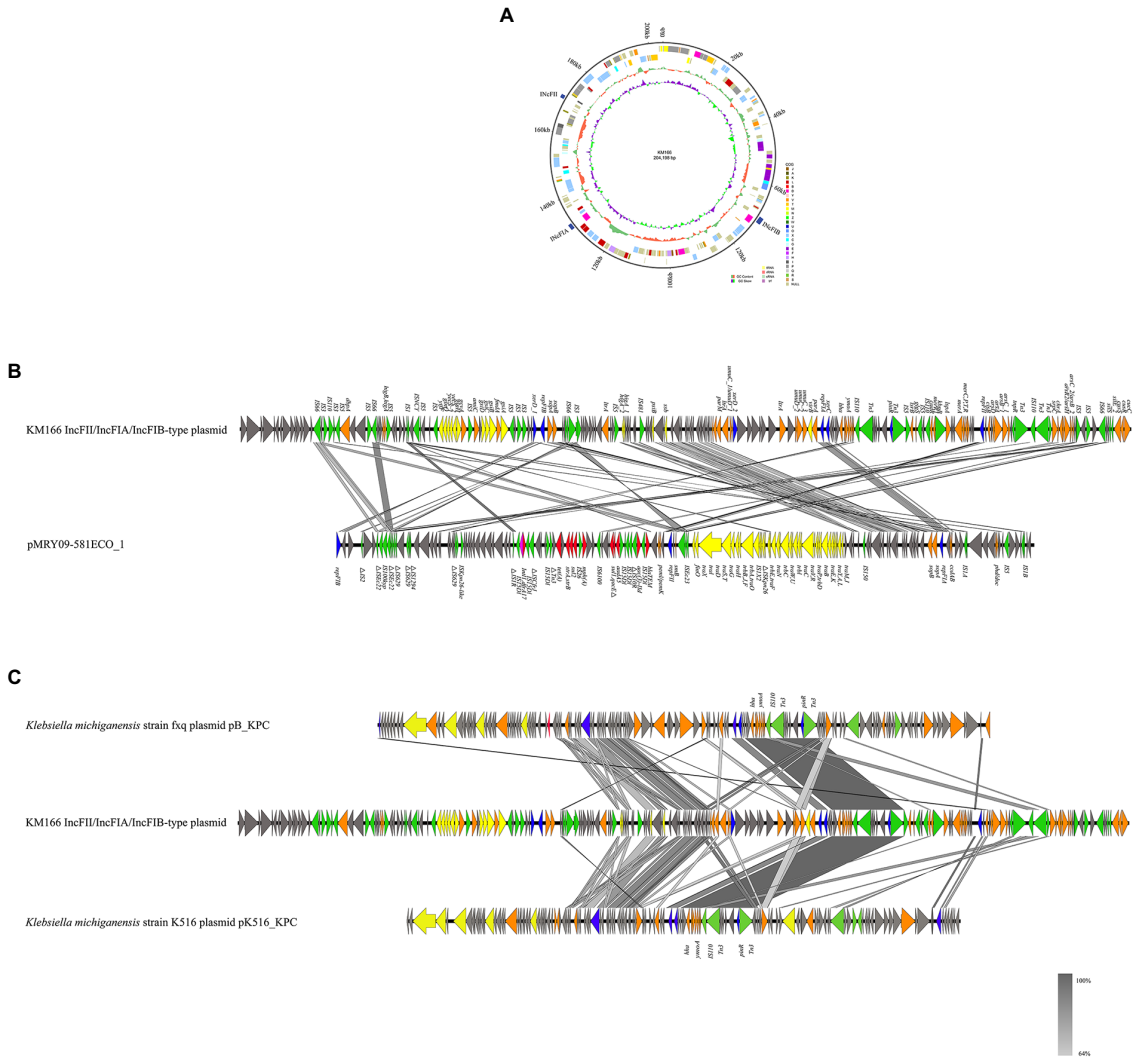
Mapping of the gene environment of a IncFII/IncFIA(HI1)/IncFIB(K) multiple replicon plasmid in *Klebsiella michiganensis* KM166. **(A)** Genome circle map of the KM166 IncFII/IncFIA(HI1)/IncFIB(K) plasmid. **(B)** Comparison of the genetic environment between KM166 IncFII/IncFIA(HI1)/IncFIB(K) plasmid and pMRY09-581ECO_1. **(C)** comparison of KM166 IncFII/IncFIA(HI1)/IncFIB(K) plasmid with the two highest homology genome sequences from NCBI. Genes involved in coupling transfer are indicated by yellow arrows, genes associated with plasmid stability and replication are highlighted in orange and blue arrows, respectively. Red and green arrows indicate antimicrobial resistance genes and mobile element genes, respectively.

We detected a 202 kb IncFII/IncFIA(HI1)/IncFIB(K) multiple replicon plasmid in KM166 Plasmid 2 (Figure 6A), which was only previously detected in *E. coli* strains from Italy and United States. A study had detected IncFII/IncFIA/IncFIB multiple replicon plasmid in 24 *E. coli* strains, the most complete sequence of which was pMRY09-581ECO_1 (accession no. AP018456), the genomic environment comparison between pMRY09-581ECO_1 and KM166 Plasmid 2 showed that the IncFII/IncFIA(HI1)/IncFIB(K) multiple replicon plasmid in KM166 was larger than pMRY09-581ECO_1, little similarities of genomic environment between them were found and many resistance genes were only detected in pMRY09-581ECO_1 (Figure 6B). Comparison of plasmid sequence with GenBank data using BLAST^3^ revealed a remarkable homology (12% coverage and 99.99% identity) to *K. michiganensis* strain fxq plasmid pB_KPC (GenBank accession no. NZ_CP095001) isolated from Zhengzhou, China in 2022 and *K. michiganensis* strain K516 plasmid pK516_KPC (15% coverage and 99.99% identity) isolated from Zhejiang, China in 2017 (GenBank accession no. NZ_CP022349). The presence of many mobile genetic elements in hha-ymoA-IS110-Tn3-pinR-Tn3 structure region of KM166 Plasmid 2 was revealed by genetic environment analysis (Figure 6C). The IncFII/IncFIA(HI1)/IncFIB(K) multiple replicon plasmid in KM166 has low homology to pMRY09-581ECO_1 and the highest homology similarity to the INcFIA/INcFII(p14)-type plasmid in *K. michiganensis* strain fxq plasmid pB_KPC, suggesting that this multiple replicon plasmid was unlikely to have been transmitted from *E. coli* and probably a transfer of *repFIB* replicon genes from other *K. michiganensis* strains into the INcFIA/INcFII(p14)-type plasmid of KM166 had occurred, resulting in a strain with multiple plasmid types co-existing.

## 4. Discussion

It is an important clinical case that a *bla*_KPC-2_*-*carrying IncFII(p14)/IncFIA plasmid and an IncFII/IncFIA(HI1)/IncFIB(K) multiple replicon plasmid were both identified in the *K. michiganensis*. In terms of clinical detection and current research condition, *K. michiganensis* is not as mature as *K. pneumoniae*, but several studies (Zheng et al., 2018; Li et al., 2021) have shown that *K. michiganensis* strains already carry multiple drug resistance genes, especially carbapenem resistance genes. *K. michiganensis* has been shown to be highly resistant and multidrug-resistant like *K. pneumoniae* (Chapman et al., 2020; Campos-Madueno et al., 2021).

There are 445 *K. michiganensis* strains in the public database, mainly from Switzerland and China. Despite the fact that the patient had not traveled abroad, it is reasonable to suspect that KM166 was transmitted from other patients with a history of international travel within the same hospital. Phylogenetic tree showed that all strains were located in one evolutionary branch, indicating that *K. michiganensis* strains have been relatively conservative in the evolution and have not undergone much evolutionary variation. The fact that *K. michiganensis* strains carry multiple drug-resistant genes serves as a reminder to us to strictly control antibiotic use in order to prevent and control healthcare associated infections.

A *bla*_KPC-2_-carrying IncFII(p14)/IncFIA plasmid carried on the *K. michiganensis* strain was rarely reported around the world. There was a 117.8-kb IncFIA-IncFII plasmid carrying the β-lactamase-encoding gene *bla*_OXA-926_ identified in a carbapenem-resistant *K. pneumoniae* strain isolated from China in 2021, which could be transferred with the facilitation of the *bla*_KPC-2-_carrying plasmid (Liu, S. et al., 2021). The IncFII(p14)/IncFIA plasmid identified in our study carried *bla*_KPC-2_ and possessed the complete conjugative regions of the self-transmissible mobile genetic elements (oriT, T4CP, T4SS), which can transfer autonomously, and the resistance genes in this plasmid could transfer with the help of mobile genetic elements around. The detection rate of carbapenem-resistant *K. pneumoniae* carrying *bla*_KPC-2_ in blood samples between 2017 and 2020 in hospitals where KM166 was isolated was 40.67% (146/359), and we therefore suspect that the *bla*_KPC-2_-carrying IncFII(p14)/IncFIA plasmid carried by KM166 may have been transferred from *K. pneumoniae* carrying *bla*_KPC-2_. Besides, it is important to recognize the significance of the *K. michiganensis* strain we isolated from a patient’s blood sample in a Beijing hospital with a total of 38 virulence genes and 41 resistance genes.

The IncFII/IncFIA(HI1)/IncFIB(K)-type plasmid has rarely been reported globally. Two *bla*_NDM-5_-carrying plasmids with IncFIA/IncFIB/IncFII/IncQ1 replicons have been found in previous studies (Hirabayashi et al., 2021; Mohamed et al., 2022). Meanwhile, some researchers discovered multiple resistance genes in an IncFII/IncFIA(HI1)/IncFIB(K) multiple replicon plasmid in *E. coli* (Hayashi et al., 2020; Marchetti et al., 2020; Simner et al., 2022). We discovered an IncFII/IncFIA(HI1)/IncFIB(K) multiple replicon plasmid in *K. michiganensis* strain KM166, implying that plasmid heterozygous transfer may be an evolutionary trend through genetic rearrangement *via* sequence insertion and homologous recombination. The IncFII/IncFIA(HI1)/IncFIB(K) multiple replicon plasmid did not contain any factors associated with autonomous transfer, nor was a resistance gene discovered, indicating that the multiple replicon plasmid we identified is still in a primitive state.

Some limitations were found in this study. Firstly, of the 445 available *K. michiganensis* strains, a relatively low percentage of isolates were from China, and the most recent source in the evolutionary analysis was a foreign strain. Expanding the number of strains collected may discover a local Chinese near-source strain. Secondly, we verified that the plasmid could be transferred from *K. michiganensis* to *E. coli*, but further validation is needed to see if the plasmid is indeed transferred from *K. pneumoniae* to *K. michiganensis*. Only 22 of the 446 *K. michiganensis* strains collected carried the *bla*_KPC-2_ gene, indicating that the *bla*_KPC-2_ carriage rate was low. The acquisition of *bla*_KPC-2_ by *K. michiganensis* strain from other *K. pneumoniae* strains may influenced by the characteristics of the strain itself and the specific environment.

In conclusion, this study described a clinical *K. michiganensis* strain carrying multiple resistance genes. The IncFII/IncFIA(HI1)/IncFIB(K) multiple replicon plasmid and *bla*_KPC-2_-carrying IncFII(p14)/IncFIA plasmid were both found in a *K. michiganensis* strain. There was probably a transfer of *repFIB* replicon genes from other *K. michiganensis* strains into the INcFIA-INcFII(p14)-type plasmid of KM166 that resulted in the occurrence of the multiple replicon IncFII/IncFIA(HI1)/IncFIB(K) plasmid. Meanwhile, the genetic environment of *bla*_KPC-2_ in IncFII(p14)/IncFIA plasmid was same as *K. pneumoniae* strain JKP55, plasmid pKPC-J5501, suggesting that the *bla*_KPC-2_ might have been transferred from *K. pneumoniae* strains under the help of mobile genetic elements. It provides a reference for understanding the clinical infection and evolutionary characteristics of *K. michiganensis*.

## Data availability statement

The datasets presented in this study can be found in online repositories. The names of the repository/repositories and accession number(s) can be found at: https://www.ncbi.nlm.nih.gov/genbank/, GCA_025263805.1.

## Ethics statement

The study was authorized by The Seven Medical Center of PLA General Hospital’s institutional ethical boards in Beijing, China (2021-82). The institutional ethics committee waived the need for informed consent from the participants because all data were gathered and analyzed anonymously.

## Author contributions

NZ and YC: conceptualization. NZ, LQ, XY, MJ, JC, SQ, and FL: data curation. NZ: formal analysis and visualization. YC, XL, and LQ: funding acquisition. NZ and JG: methodology. YC: project administration. NZ and XL: software. CW and YC: supervision. NZ, XL, and LQ: writing—original draft. NZ, XL, and YC: writing—review and editing. All authors contributed to the article and approved the submitted version.

## Funding

The work was supported by a grant from National Key Program for Infectious Diseases of China (2018ZX10733-402), Beijing Nova Program (Z181100006218107), National Natural Science Foundation of China (31900151), National Natural Science Foundation of China (81902112).

## Conflict of interest

The authors declare that the research was conducted in the absence of any commercial or financial relationships that could be construed as a potential conflict of interest.

## Publisher’s note

All claims expressed in this article are solely those of the authors and do not necessarily represent those of their affiliated organizations, or those of the publisher, the editors and the reviewers. Any product that may be evaluated in this article, or claim that may be made by its manufacturer, is not guaranteed or endorsed by the publisher.

## Footnotes

^1^http://phast.wishartlab.com

^2^ https://crisprcas.i2bc.paris-saclay.fr/CrisprCasFinder/Index

^3^ https://blast.ncbi.nlm.nih.gov/Blast.cgi

## References

[ref1] Abed J. Y. Déraspe M. Bérubé È. D'Iorio M. Dewar K. Boissinot M.. (2021). Complete genome sequences of Klebsiella michiganensis and Citrobacter farmeri, KPC-2-producers serially isolated from a single patient. Antibiotics (Basel). 10:1408. doi: 10.3390/antibiotics10111408, PMID: 34827346 PMC8614947

[ref2] Alcock B. P. Raphenya A. R. Lau T. T. Y. Tsang K. K. Bouchard M. Edalatmand A.. (2020). CARD 2020: antibiotic resistome surveillance with the comprehensive antibiotic resistance database. Nucleic Acids Res. 48, D517–D525. doi: 10.1093/nar/gkz935, PMID: 31665441 PMC7145624

[ref3] Cai J. C. Zhou H. W. Zhang R. Chen G. X. (2008). Emergence of Serratia marcescens, Klebsiella pneumoniae, and Escherichia coli isolates possessing the plasmid-mediated carbapenem-hydrolyzing beta-lactamase KPC-2 in intensive care units of a Chinese hospital. Antimicrob. Agents Chemother. 52, 2014–2018. doi: 10.1128/AAC.01539-07, PMID: 18332176 PMC2415814

[ref4] Campos-Madueno E. I. Sigrist T. Flückiger U. M. Risch L. Bodmer T. Endimiani A. (2021). First report of a Bla(VIM-1) metallo-β-lactamase-possessing Klebsiella michiganensis. J Glob Antimicrob Resist. 25, 310–314. doi: 10.1016/j.jgar.2021.03.027, PMID: 33957287

[ref5] Catalán-Nájera J. C. Garza-Ramos U. Barrios-Camacho H. (2017). Hypervirulence and hypermucoviscosity: two different but complementary Klebsiella spp. phenotypes? Virulence 8, 1111–1123. doi: 10.1080/21505594.2017.1317412, PMID: 28402698 PMC5711391

[ref6] Ccorahua-Santo R. Cervantes M. Duran Y. Aguirre M. Marin C. Ramírez P. (2017). Draft genome sequence of Klebsiella michiganensis 3T412C, harboring an arsenic resistance Genomic Island, isolated from mine tailings in Peru. Genome Announc. 5:0611. doi: 10.1128/genomeA.00611-17, PMID: 28705974 PMC5511913

[ref7] Chan P. A.-O. Lin B. Y. Mak A. J. Lowe T. A.-O. (2021). tRNAscan-SE 2.0: improved detection and functional classification of transfer RNA genes. Nucleic Acids Res. 49, 9077–9096. doi: 10.1093/nar/gkab688, PMID: 34417604 PMC8450103

[ref8] Chapman P. Forde B. M. Roberts L. W. Bergh H. Vesey D. Jennison A. V.. (2020). Genomic investigation reveals contaminated detergent as the source of an extended-Spectrum-β-lactamase-producing Klebsiella michiganensis outbreak in a neonatal unit. J. Clin. Microbiol. 58:980. doi: 10.1128/jcm.01980-19, PMID: 32102855 PMC7180233

[ref9] Darriba D. Posada D. Kozlov A. M. Stamatakis A. Morel B. Flouri T. (2020). ModelTest-NG: a new and scalable tool for the selection of DNA and protein evolutionary models. Mol. Biol. Evol. 37, 291–294. doi: 10.1093/molbev/msz189, PMID: 31432070 PMC6984357

[ref10] Didelot X. Wilson D. J. (2015). ClonalFrameML: efficient inference of recombination in whole bacterial genomes. PLoS Comput. Biol. 11:e1004041. doi: 10.1371/journal.pcbi.1004041, PMID: 25675341 PMC4326465

[ref11] Doi Y. Paterson D. L. (2015). Carbapenemase-producing Enterobacteriaceae. Semin. Respir. Crit. Care Med. 36, 74–84. doi: 10.1055/s-0035-1544208, PMID: 25643272 PMC4470611

[ref12] Flouri T. Izquierdo-Carrasco F. Darriba D. Aberer A. J. Nguyen L. T. Minh B. Q.. (2015). The phylogenetic likelihood library. Syst. Biol. 64, 356–362. doi: 10.1093/sysbio/syu084, PMID: 25358969 PMC4380035

[ref13] Founou R. C. Founou L. L. Allam M. Ismail A. Essack S. Y. (2018). Genomic characterisation of Klebsiella michiganensis co-producing OXA-181 and NDM-1 carbapenemases isolated from a cancer patient in uMgungundlovu district, KwaZulu-Natal Province. South Africa. S Afr Med J. 109, 7–8. doi: 10.7196/SAMJ.2018.v109i1.13696, PMID: 30606295

[ref14] Gu D. Dong N. Zheng Z. Lin D. Huang M. Wang L.. (2018). A fatal outbreak of ST11 carbapenem-resistant hypervirulent Klebsiella pneumoniae in a Chinese hospital: a molecular epidemiological study. Lancet Infect. Dis. 18, 37–46. doi: 10.1016/S1473-3099(17)30489-9, PMID: 28864030

[ref15] Hayashi M. Matsui M. Sekizuka T. Shima A. Segawa T. Kuroda M.. (2020). Dissemination of IncF group F1:A2:B20 plasmid-harbouring multidrug-resistant Escherichia coli ST131 before the acquisition of bla_CTX-M_ in Japan. J Glob Antimicrob Resist. 23, 456–465. doi: 10.1016/j.jgar.2020.10.021, PMID: 33212283

[ref16] Hirabayashi A. Yanagisawa H. Takahashi H. Yahara K. Boeing P. Wolfenden B.. (2021). On-site genomic epidemiological analysis of antimicrobial-resistant bacteria in Cambodia with portable laboratory equipment. Front. Microbiol. 12:675463. doi: 10.3389/fmicb.2021.675463, PMID: 34054783 PMC8158813

[ref17] Iovleva A. Doi Y. (2017). Carbapenem-Resistant Enterobacteriaceae. Clin. Lab. Med. 37, 303–315. doi: 10.1016/j.cll.2017.01.005, PMID: 28457352 PMC5412586

[ref18] Kang M. Chmara J. Duceppe M. O. Phipps-Todd B. Huang H. (2020). Complete genome sequence of a Canadian Klebsiella michiganensis strain, obtained using Oxford Nanopore technologies sequencing. Microbiol Resour Announc. 9:960. doi: 10.1128/mra.00960-20, PMID: 33184156 PMC7660995

[ref19] King T. L. Schmidt S. Thakur S. Fedorka-Cray P. Keelara S. Harden L.. (2021). Resistome of a carbapenemase-producing novel ST232 Klebsiella michiganensis isolate from urban hospital effluent in South Africa. J Glob Antimicrob Resist. 24, 321–324. doi: 10.1016/j.jgar.2021.01.004, PMID: 33508483 PMC8012231

[ref20] Koren S. Walenz B. P. Berlin K. Miller J. R. Bergman N. H. Phillippy A. M. (2017). Canu: scalable and accurate long-read assembly via adaptive k-mer weighting and repeat separation. Genome Res. 27, 722–736. doi: 10.1101/gr.215087.116, PMID: 28298431 PMC5411767

[ref21] Kozlov A. M. Darriba D. Flouri T. Morel B. Stamatakis A. (2019). RAxML-NG: a fast, scalable and user-friendly tool for maximum likelihood phylogenetic inference. Bioinformatics 35, 4453–4455. doi: 10.1093/bioinformatics/btz305, PMID: 31070718 PMC6821337

[ref22] Lam M. Wick R. R. Watts S. C. Cerdeira L. T. Wyres K. L. Holt K. E. (2021). A genomic surveillance framework and genotyping tool for Klebsiella pneumoniae and its related species complex. Nat. Commun. 12:4188. doi: 10.1038/s41467-021-24448-3, PMID: 34234121 PMC8263825

[ref23] Letunic I. Bork P. (2021). Interactive tree of life (iTOL) v5: an online tool for phylogenetic tree display and annotation. Nucleic Acids Res. 49, W293–W296. doi: 10.1093/nar/gkab301, PMID: 33885785 PMC8265157

[ref24] Li X. He J. Jiang Y. Peng M. Yu Y. Fu Y. (2021). Genetic characterization and passage instability of a hybrid plasmid co-harboring Bla(IMP-4) and Bla(NDM-1) reveal the contribution of insertion sequences during plasmid formation and evolution. Microbiol Spectr 9:e0157721. doi: 10.1128/Spectrum.01577-21, PMID: 34908434 PMC8672901

[ref25] Liu L. Feng Y. Wei L. Xiao Y. Zong Z. (2021). KPC-2-producing Carbapenem-resistant Klebsiella pneumoniae of the uncommon ST29 type carrying OXA-926, a novel narrow-Spectrum OXA β-lactamase. Front. Microbiol. 12:701513. doi: 10.3389/fmicb.2021.701513, PMID: 34512578 PMC8430219

[ref26] Liu S. Liu H. Chen R. Ma Y. Yang B. Chen Z.. (2021). Role of two plant growth-promoting bacteria in remediating cadmium-contaminated soil combined with Miscanthus floridulus (lab.). Plants (Basel). 10:912. doi: 10.3390/plants10050912, PMID: 34063227 PMC8147505

[ref27] Liu Y. Long D. Xiang T. X. Du F. L. Wei D. D. Wan L. G.. (2019). Whole genome assembly and functional portrait of hypervirulent extensively drug-resistant NDM-1 and KPC-2 co-producing Klebsiella pneumoniae of capsular serotype K2 and ST86. J. Antimicrob. Chemother. 74, 1233–1240. doi: 10.1093/jac/dkz023, PMID: 30843067

[ref28] Liu B. Zheng D. Jin Q. Chen L. Yang J. (2019). VFDB 2019: a comparative pathogenomic platform with an interactive web interface. Nucleic Acids Res. 47, D687–D692. doi: 10.1093/nar/gky1080, PMID: 30395255 PMC6324032

[ref29] Luo X. Zhang J. Yuan M. Mou S. Xu M. Hu D.. (2022). Epidemiology of Klebsiella michiganensis carrying multidrug-resistant IncHI5 plasmids in the southeast coastal area of China. Infect Drug Resist. 15, 1831–1843. doi: 10.2147/idr.S358839, PMID: 35444429 PMC9013925

[ref30] Marchetti V. M. Bitar I. Mercato A. Nucleo E. Bonomini A. Pedroni P.. (2020). Complete nucleotide sequence of plasmids of two Escherichia coli strains carrying bla_NDM-5_ and bla_NDM-5_ and bla_OXA-181_ from the same patient. Front. Microbiol. 10:3095. doi: 10.3389/fmicb.2019.03095, PMID: 32038543 PMC6985152

[ref31] Mitra S. Pramanik K. Ghosh P. K. Soren T. Sarkar A. Dey R. S.. (2018). Characterization of cd-resistant Klebsiella michiganensis MCC3089 and its potential for rice seedling growth promotion under cd stress. Microbiol. Res. 210, 12–25. doi: 10.1016/j.micres.2018.03.003, PMID: 29625654

[ref32] Mitra S. Purkait T. Pramanik K. Maiti T. K. Dey R. S. (2019). Three-dimensional graphene for electrochemical detection of cadmium in Klebsiella michiganensis to study the influence of cadmium uptake in rice plant. Mater. Sci. Eng. C Mater. Biol. Appl. 103:109802. doi: 10.1016/j.msec.2019.109802, PMID: 31349442

[ref33] Mohamed N. M. Zakaria A. S. Edward E. A. (2022). Genomic characterization of international high-risk clone ST410 Escherichia coli co-harboring ESBL-encoding genes and bla_NDM-5_ on IncFIA/IncFIB/IncFII/IncQ1 multireplicon plasmid and carrying a chromosome-borne bla_CMY-2_ from Egypt. Antibiotics (Basel). 11:1031. doi: 10.3390/antibiotics11081031, PMID: 36009900 PMC9405272

[ref34] Nordmann P. Naas T. Poirel L. (2011). Global spread of Carbapenemase-producing Enterobacteriaceae. Emerg. Infect. Dis. 17, 1791–1798. doi: 10.3201/eid1710.110655, PMID: 22000347 PMC3310682

[ref35] Oinuma K. I. Suzuki M. A.-O. Sakiyama A. Tsubouchi T. Saeki K. Sato K.. (2021). Genomic characterization of triple-carbapenemase-producing Acinetobacter baumannii. JAC Antimicrob Resist. 3:dlab191. doi: 10.1093/jacamr/dlab191, PMID: 34934945 PMC8684466

[ref36] Oliveira R. A. Ng K. M. Correia M. B. Cabral V. Shi H. Sonnenburg J. L.. (2020). Klebsiella michiganensis transmission enhances resistance to Enterobacteriaceae gut invasion by nutrition competition. Nat. Microbiol. 5, 630–641. doi: 10.1038/s41564-019-0658-4, PMID: 31959968

[ref37] Pitout J. D. Nordmann P. Poirel L. (2015). Carbapenemase-producing Klebsiella pneumoniae, a key pathogen set for global nosocomial dominance. Antimicrob. Agents Chemother. 59, 5873–5884. doi: 10.1128/AAC.01019-15, PMID: 26169401 PMC4576115

[ref38] Salgueiro V. Manageiro V. Bandarra N. M. Reis L. Ferreira E. Caniça M. (2020). Bacterial diversity and antibiotic susceptibility of Sparus aurata from aquaculture. Microorganisms. 8:343. doi: 10.3390/microorganisms8091343, PMID: 32887439 PMC7564983

[ref39] Seemann T. (2014). Prokka: rapid prokaryotic genome annotation. Bioinformatics 30, 2068–2069. doi: 10.1093/bioinformatics/btu153, PMID: 24642063

[ref40] Seiffert S. N. Wüthrich D. Gerth Y. Egli A. Kohler P. Nolte O. (2019). First clinical case of KPC-3-producing Klebsiella michiganensis in Europe. New Microbes New Infect. 29:100516. doi: 10.1016/j.nmni.2019.100516, PMID: 30949345 PMC6428935

[ref41] Simner P. J. Mostafa H. H. Bergman Y. Ante M. Tekle T. Adebayo A.. (2022). Progressive development of Cefiderocol resistance in Escherichia coli during therapy is associated with an increase in bla_NDM-5_ copy number and gene expression. Clin. Infect. Dis. 75, 47–54. doi: 10.1093/cid/ciab888, PMID: 34618008 PMC9402677

[ref42] Siu L. K. Fung C. P. Chang F. Y. Lee N. Yeh K. M. Koh T. H.. (2011). Molecular typing and virulence analysis of serotype K1 Klebsiella pneumoniae strains isolated from liver abscess patients and stool samples from noninfectious subjects in Hong Kong, Singapore, and Taiwan. J. Clin. Microbiol. 49, 3761–3765. doi: 10.1128/JCM.00977-11, PMID: 21900521 PMC3209116

[ref43] Sullivan M. J. Petty N. K. Beatson S. A. (2011). Easyfig: a genome comparison visualizer. Bioinformatics 27, 1009–1010. doi: 10.1093/bioinformatics/btr039, PMID: 21278367 PMC3065679

[ref44] Wyres K. L. Lam M. Holt K. E. (2020). Population genomics of Klebsiella pneumoniae. Nat. Rev. Microbiol. 18, 344–359. doi: 10.1038/s41579-019-0315-1 32055025

[ref45] Xu J. Ding H. Zhao Y. Chen J. Zhao Z. Huang J.. (2022). Characterization of a novel Carbapenem-resistant Klebsiella michiganensis strain Coharboring the Bla(SIM-1), Bla(OXA-1), Bla(CTX-M-14), qnrS, and aac(6′)-Ib-cr genes. Curr. Microbiol. 79:228. doi: 10.1007/s00284-022-02920-7, PMID: 35751714

[ref46] Yan X. Yang J. Wang Q. Lin S. (2021). Transcriptomic analysis reveals resistance mechanisms of Klebsiella michiganensis to copper toxicity under acidic conditions. Ecotoxicol. Environ. Saf. 211:111919. doi: 10.1016/j.ecoenv.2021.111919, PMID: 33476853

[ref47] Zhang Y. Gu D. Yang X. Wu Y. Liu C. Shen Z.. (2021). Emergence and genomic characterization of a KPC-2-, NDM-1-, and IMP-4-producing Klebsiella michiganensis isolate. Front. Microbiol. 12:762509. doi: 10.3389/fmicb.2021.762509, PMID: 35069468 PMC8770907

[ref48] Zheng B. Xu H. Yu X. Lv T. Jiang X. Cheng H.. (2018). Identification and genomic characterization of a KPC-2-, NDM-1- and NDM-5-producing Klebsiella michiganensis isolate. J. Antimicrob. Chemother. 73, 536–538. doi: 10.1093/jac/dkx415, PMID: 29126236

