# Supplementary material for: A clinical KPC-producing Klebsiella michiganensis strain carrying IncFII/IncFIA (HI1)/IncFIB (K) multiple replicon plasmid
Source: Front Microbiol. 2023 Jan 4;13:1086296. doi: 10.3389/fmicb.2022.1086296 (PMC9845883; doi:10.3389/fmicb.2022.1086296)
Supplement: Supplementary file 1 [file Table_1.DOCX]

Supplementary Material

# Supplementary Tables

## Table S1 Genomic information of 445 *Klebsiella michiganensis* strains downloaded from Pathosystems Resource Integration Center.

| PATRIC Name | Genome Name | Genome Status | BioProject Accession | BioSample Accession | Genome Length(bp) | GC Content(%) |
| --- | --- | --- | --- | --- | --- | --- |
| 1006551.4 | *Klebsiella oxytoca* KCTC 1686 | Complete | PRJNA65523 | SAMN02603580 | 5974109 | 56.05 |
| 1134687.133 | *Klebsiella michiganensis* strain M82255 | Complete | PRJNA512395 | SAMN10743308 | 6604517 | 55.51 |
| 1134687.134 | *Klebsiella michiganensis* strain A202R3B6 | WGS | PRJNA429538 | SAMN09396863 | 6402553 | 55.09 |
| 1134687.135 | *Klebsiella michiganensis* strain Kox37 | WGS | PRJNA511522 | SAMN10639457 | 6333233 | 55.44 |
| 1134687.146 | *Klebsiella michiganensis* strain SDF3 | WGS | PRJNA552487 | SAMN12211897 | 5855606 | 55.99 |
| 1134687.147 | *Klebsiella michiganensis* strain SDG3 | WGS | PRJNA552487 | SAMN12211898 | 5856227 | 55.99 |
| 1134687.148 | *Klebsiella michiganensis* strain KNU07 | Complete | PRJNA553277 | SAMN12233455 | 6270897 | 55.77 |
| 1134687.149 | *Klebsiella michiganensis* strain P095L Y | WGS | PRJNA471164 | SAMN09204099 | 6292440 | 55.48 |
| 1134687.150 | *Klebsiella michiganensis* strain P049A W | WGS | PRJNA471164 | SAMN09204079 | 6192097 | 55.80 |
| 1134687.151 | *Klebsiella michiganensis* strain T0101B.F-25 | WGS | PRJNA489090 | SAMN09981286 | 6266224 | 55.83 |
| 1134687.152 | *Klebsiella michiganensis* strain FDAARGOS_647 strain Not applicable | Complete | PRJNA231221 | SAMN11056362 | 6656590 | 55.57 |
| 1134687.153 | *Klebsiella michiganensis* strain SB9 | WGS | PRJEB15325 | SAMEA5610065 | 6191219 | 55.67 |
| 1134687.154 | *Klebsiella michiganensis* strain SB4934 | WGS | PRJEB15325 | SAMEA5610064 | 6193009 | 55.97 |
| 1134687.156 | *Klebsiella michiganensis* strain SB2908 | WGS | PRJEB15325 | SAMEA5610066 | 5989425 | 55.70 |
| 1134687.16 | *Klebsiella michiganensis* strain 3T412C | WGS | PRJNA352878 | SAMN06006480 | 6208338 | 55.72 |
| 1134687.161 | *Klebsiella michiganensis* strain MGYG-HGUT-02351 | WGS | PRJEB33885 | SAMEA5851855 | 6684898 | 55.65 |
| 1134687.163 | *Klebsiella michiganensis* strain C52 | Complete | PRJNA550014 | SAMN12289379 | 6312326 | 55.85 |
| 1134687.164 | *Klebsiella michiganensis* strain CCUG 66515 | WGS | PRJNA563568 | SAMN12697578 | 6156948 | 55.99 |
| 1134687.166 | *Klebsiella michiganensis* strain 1305118 | WGS | PRJNA597427 | SAMN13675360 | 5767757 | 56.04 |
| 1134687.167 | *Klebsiella michiganensis* strain F107 | Complete | PRJNA395651 | SAMN07411959 | 6152545 | 55.38 |
| 1134687.17 | *Klebsiella michiganensis* strain MGH175 | WGS | PRJNA271899 | SAMN04521924 | 6438325 | 55.27 |
| 1134687.176 | *Klebsiella michiganensis* strain BD177 | Complete | PRJNA602959 | SAMN13911826 | 6812698 | 55.04 |
| 1134687.177 | *Klebsiella michiganensis* strain AS012494 | WGS | PRJNA553678 | SAMN12250813 | 6037619 | 55.99 |
| 1134687.178 | *Klebsiella michiganensis* strain AS012477 | WGS | PRJNA553678 | SAMN12250796 | 6316346 | 55.94 |
| 1134687.179 | *Klebsiella michiganensis* strain AS012469 | WGS | PRJNA553678 | SAMN12250788 | 6250319 | 55.91 |
| 1134687.18 | *Klebsiella michiganensis* strain MGH176 | WGS | PRJNA271899 | SAMN04521925 | 6147269 | 54.72 |
| 1134687.180 | *Klebsiella michiganensis* strain AS012456 | WGS | PRJNA553678 | SAMN12250775 | 6259934 | 55.82 |
| 1134687.181 | *Klebsiella michiganensis* strain AS012446 | WGS | PRJNA553678 | SAMN12250765 | 6042893 | 56.07 |
| 1134687.184 | *Klebsiella michiganensis* strain Biosolid 38 | WGS | PRJNA609701 | SAMN14258347 | 6450309 | 55.52 |
| 1134687.185 | *Klebsiella michiganensis* strain AS012270 | WGS | PRJNA553678 | SAMN12250589 | 6221169 | 55.95 |
| 1134687.186 | *Klebsiella michiganensis* strain JH07 | WGS | PRJNA615066 | SAMN14604412 | 5899769 | 55.92 |
| 1134687.192 | *Klebsiella michiganensis* strain CRN 27 | WGS | PRJNA622881 | SAMN14531576 | 5841460 | 55.98 |
| 1134687.194 | *Klebsiella michiganensis* strain Biosolid 27 | Complete | PRJNA609701 | SAMN14258344 | 5891206 | 56.07 |
| 1134687.195 | *Klebsiella michiganensis* strain ZH142-C | WGS | PRJNA603881 | SAMN13943465 | 6377024 | 55.66 |
| 1134687.199 | *Klebsiella michiganensis* strain RHBSTW-00167 | Complete | PRJNA605147 | SAMN15148522 | 6544295 | 55.48 |
| 1134687.200 | *Klebsiella michiganensis* strain RHB20-C02 | Complete | PRJNA605147 | SAMN15148174 | 6236062 | 55.54 |
| 1134687.201 | *Klebsiella michiganensis* strain RHBSTW-00909 | Complete | PRJNA605147 | SAMN15148741 | 6609426 | 55.14 |
| 1134687.202 | *Klebsiella michiganensis* strain F52 | WGS | PRJNA609897 | SAMN14262042 | 6253068 | 55.73 |
| 1134687.203 | *Klebsiella michiganensis* strain RHBSTW-00676 | Complete | PRJNA605147 | SAMN15148681 | 6195779 | 55.58 |
| 1134687.204 | *Klebsiella michiganensis* strain PS_Koxy2 | WGS | PRJNA562720 | SAMN12648329 | 6275379 | 55.75 |
| 1134687.205 | *Klebsiella michiganensis* strain PS_Koxy4 | WGS | PRJNA562720 | SAMN12648330 | 6294880 | 55.73 |
| 1134687.206 | *Klebsiella michiganensis* strain PS_Koxy1 | WGS | PRJNA562720 | SAMN12648328 | 6271778 | 55.73 |
| 1134687.207 | *Klebsiella michiganensis* strain RHBSTW-00900 | WGS | PRJNA605147 | SAMN15148734 | 6649205 | 55.37 |
| 1134687.208 | *Klebsiella michiganensis* strain RHBSTW-00109 | WGS | PRJNA605147 | SAMN15148490 | 6337052 | 55.57 |
| 1134687.209 | *Klebsiella michiganensis* strain RHB20-C01 | WGS | PRJNA605147 | SAMN15148173 | 6240015 | 55.53 |
| 1134687.21 | *Klebsiella michiganensis* strain DPB_3 | WGS | PRJNA184698 | SAMN06009455 | 6582107 | 55.55 |
| 1134687.210 | *Klebsiella michiganensis* strain ZLTJD1 | WGS | PRJNA647063 | SAMN15576906 | 5822565 | 55.88 |
| 1134687.211 | *Klebsiella michiganensis* strain AKKL-001 | Complete | PRJNA655785 | SAMN15755083 | 6149586 | 55.83 |
| 1134687.214 | *Klebsiella michiganensis* strain Kop | WGS | PRJNA656584 | SAMN15634860 | 5812670 | 55.68 |
| 1134687.215 | *Klebsiella michiganensis* strain EC608 | WGS | PRJNA528851 | SAMN11246454 | 6060440 | 56.08 |
| 1134687.216 | *Klebsiella michiganensis* strain SCKM090650 | WGS | PRJNA353728 | SAMN14379875 | 6503826 | 55.67 |
| 1134687.217 | *Klebsiella michiganensis* strain SCKM090647 | WGS | PRJNA353728 | SAMN14379873 | 6333510 | 55.68 |
| 1134687.218 | *Klebsiella michiganensis* strain SCKM090640 | WGS | PRJNA353728 | SAMN14379872 | 6294041 | 55.16 |
| 1134687.219 | *Klebsiella michiganensis* strain SCKM090631 | WGS | PRJNA353728 | SAMN14379871 | 6534624 | 55.37 |
| 1134687.22 | *Klebsiella michiganensis* strain K516 | Complete | PRJNA393093 | SAMN07315162 | 6418740 | 55.83 |
| 1134687.220 | *Klebsiella michiganensis* strain SCKM090630 | WGS | PRJNA353728 | SAMN14379870 | 6641954 | 54.96 |
| 1134687.221 | *Klebsiella michiganensis* strain KO13459-1 | WGS | PRJNA552260 | SAMN12219632 | 6516012 | 55.38 |
| 1134687.222 | *Klebsiella michiganensis* strain KO16290 | WGS | PRJNA552260 | SAMN12220030 | 5981743 | 55.58 |
| 1134687.223 | *Klebsiella michiganensis* strain KO17045 | WGS | PRJNA552260 | SAMN12220031 | 5983577 | 55.60 |
| 1134687.224 | *Klebsiella michiganensis* strain KO14657 | WGS | PRJNA552260 | SAMN12220020 | 6240538 | 55.54 |
| 1134687.225 | *Klebsiella michiganensis* strain KO16162 | WGS | PRJNA552260 | SAMN12220021 | 6226931 | 55.54 |
| 1134687.226 | *Klebsiella michiganensis* strain KO13048 | WGS | PRJNA552260 | SAMN12220017 | 6232493 | 55.54 |
| 1134687.227 | *Klebsiella michiganensis* strain KO14641 | WGS | PRJNA552260 | SAMN12220019 | 6241139 | 55.54 |
| 1134687.228 | *Klebsiella michiganensis* strain KO13137a | WGS | PRJNA552260 | SAMN12220018 | 6216542 | 55.55 |
| 1134687.229 | *Klebsiella michiganensis* strain KO13047 | WGS | PRJNA552260 | SAMN12220016 | 6226333 | 55.55 |
| 1134687.23 | *Klebsiella michiganensis* strain K1439 | WGS | PRJNA393804 | SAMN07344979 | 6135809 | 55.90 |
| 1134687.230 | *Klebsiella michiganensis* strain THO-011 | Complete | PRJDB9036 | SAMD00196009 | 6041841 | 55.99 |
| 1134687.231 | *Klebsiella michiganensis* strain CPE6 | WGS | PRJNA662907 | SAMN16125014 | 6156021 | 55.47 |
| 1134687.232 | *Klebsiella michiganensis* strain CPE5 | WGS | PRJNA662907 | SAMN16125013 | 6274028 | 55.40 |
| 1134687.233 | *Klebsiella michiganensis* strain 1001254B_151014_G7 | WGS | PRJNA637878 | SAMN15532884 | 6005414 | 55.91 |
| 1134687.235 | *Klebsiella michiganensis* strain 401065-17 | WGS | PRJNA543274 | SAMN12212153 | 5944585 | 55.91 |
| 1134687.237 | *Klebsiella michiganensis* strain KLO00038 | WGS | PRJNA475751 | SAMN16340196 | 6033045 | 55.96 |
| 1134687.238 | *Klebsiella michiganensis* strain KLO00030 | WGS | PRJNA475751 | SAMN12220379 | 5930422 | 56.07 |
| 1134687.239 | *Klebsiella michiganensis* strain KLO00031 | WGS | PRJNA475751 | SAMN12220380 | 5939882 | 56.07 |
| 1134687.24 | *Klebsiella michiganensis* strain K518 | Complete | PRJNA393093 | SAMN07551913 | 6418567 | 55.83 |
| 1134687.240 | *Klebsiella michiganensis* strain KLO00029 | WGS | PRJNA475751 | SAMN12220378 | 6034639 | 55.89 |
| 1134687.241 | *Klebsiella michiganensis* strain KLO00019 | WGS | PRJNA475751 | SAMN11285031 | 6186625 | 55.64 |
| 1134687.242 | *Klebsiella michiganensis* strain KLO00023 | WGS | PRJNA475751 | SAMN11285033 | 6503460 | 55.91 |
| 1134687.243 | *Klebsiella michiganensis* strain KLO00020 | WGS | PRJNA475751 | SAMN11285032 | 6245529 | 55.72 |
| 1134687.244 | *Klebsiella michiganensis* strain KLO00018 | WGS | PRJNA475751 | SAMN10435694 | 6049027 | 56.01 |
| 1134687.245 | *Klebsiella michiganensis* strain KLO00016 | WGS | PRJNA475751 | SAMN10435692 | 6891268 | 55.42 |
| 1134687.246 | *Klebsiella michiganensis* strain KLO00015 | WGS | PRJNA475751 | SAMN10435691 | 7004956 | 55.37 |
| 1134687.247 | *Klebsiella michiganensis* strain KLO00012 | WGS | PRJNA475751 | SAMN09400890 | 6051383 | 55.97 |
| 1134687.248 | *Klebsiella michiganensis* strain KLO00013 | WGS | PRJNA475751 | SAMN09400891 | 6074727 | 56.01 |
| 1134687.249 | *Klebsiella michiganensis* strain KLO00011 | WGS | PRJNA475751 | SAMN09400889 | 6047686 | 55.96 |
| 1134687.250 | *Klebsiella michiganensis* strain KLO00010 | WGS | PRJNA475751 | SAMN09400888 | 6045843 | 55.97 |
| 1134687.251 | *Klebsiella michiganensis* strain KLO00005 | WGS | PRJNA475751 | SAMN09400884 | 6421054 | 55.63 |
| 1134687.253 | *Klebsiella michiganensis* strain Lac4 | WGS | PRJNA681464 | SAMN16954134 | 6359207 | 55.86 |
| 1134687.254 | *Klebsiella michiganensis* strain 7525 | Complete | PRJNA681062 | SAMN16931445 | 6244801 | 55.27 |
| 1134687.258 | *Klebsiella michiganensis* strain BD-50-Km | Complete | PRJNA664790 | SAMN16233476 | 6865058 | 55.48 |
| 1134687.259 | *Klebsiella michiganensis* strain H08 | WGS | PRJNA688543 | SAMN17180721 | 6082645 | 55.64 |
| 1134687.260 | *Klebsiella michiganensis* strain KA43_C | WGS | PRJNA480723 | SAMN13301616 | 6288658 | 55.51 |
| 1134687.261 | *Klebsiella michiganensis* strain KO99_C | WGS | PRJNA480723 | SAMN13301612 | 5750682 | 56.15 |
| 1134687.263 | *Klebsiella michiganensis* strain KO66_H | WGS | PRJNA480723 | SAMN13301610 | 6155687 | 55.86 |
| 1134687.264 | *Klebsiella michiganensis* strain KO12_C | WGS | PRJNA480723 | SAMN13301602 | 6036410 | 56.00 |
| 1134687.265 | *Klebsiella michiganensis* strain KO70_H | WGS | PRJNA480723 | SAMN13301611 | 6086676 | 56.00 |
| 1134687.266 | *Klebsiella michiganensis* strain KO36_C | WGS | PRJNA480723 | SAMN13301605 | 6166191 | 55.79 |
| 1134687.267 | *Klebsiella michiganensis* strain KO7_H | WGS | PRJNA480723 | SAMN13301600 | 6191274 | 55.88 |
| 1134687.268 | *Klebsiella michiganensis* strain KLEB011 | WGS | PRJNA690982 | SAMN17272039 | 6092037 | 55.68 |
| 1134687.269 | *Klebsiella michiganensis* strain KLEB016 | WGS | PRJNA690982 | SAMN17319808 | 6422843 | 55.72 |
| 1134687.270 | *Klebsiella michiganensis* strain KLEB013 | WGS | PRJNA690982 | SAMN17319805 | 5591412 | 56.13 |
| 1134687.271 | *Klebsiella michiganensis* strain KLEB015 | WGS | PRJNA690982 | SAMN17319807 | 6184011 | 55.77 |
| 1134687.272 | *Klebsiella michiganensis* strain KLEB014 | WGS | PRJNA690982 | SAMN17319806 | 6127656 | 55.94 |
| 1134687.273 | *Klebsiella michiganensis* strain CPO116 | WGS | PRJNA693599 | SAMN17487922 | 6496134 | 55.45 |
| 1134687.274 | *Klebsiella michiganensis* strain KO46817 | WGS | PRJNA700516 | SAMN16933264 | 6310234 | 56.00 |
| 1134687.279 | *Klebsiella michiganensis* strain KO45182 | Complete | PRJNA700516 | SAMN16933258 | 6636409 | 55.75 |
| 1134687.280 | *Klebsiella michiganensis* strain Kmfe267 | Complete | PRJNA638288 | SAMN18138829 | 6398286 | 55.52 |
| 1134687.281 | *Klebsiella michiganensis* strain 12084 | Complete | PRJNA716129 | SAMN18395722 | 6248475 | 55.80 |
| 1134687.282 | *Klebsiella michiganensis* strain LDS17 | Complete | PRJNA681064 | SAMN16931446 | 5791694 | 56.12 |
| 1134687.283 | *Klebsiella michiganensis* strain ET24 | WGS | PRJNA634137 | SAMN16729814 | 6033822 | 56.10 |
| 1134687.284 | *Klebsiella michiganensis* strain X2-1 | Complete | PRJNA624816 | SAMN14585699 | 5845628 | 55.83 |
| 1134687.290 | *Klebsiella michiganensis* strain 20-MO00090-0 | WGS | PRJNA633320 | SAMN14941265 | 6416870 | 55.75 |
| 1134687.291 | *Klebsiella michiganensis* strain 20-MO00090-1 | WGS | PRJNA633321 | SAMN14941266 | 6411472 | 55.75 |
| 1134687.292 | *Klebsiella michiganensis* strain SCPM-O-B-9255 (F28-4Ko/19) strain SCPM-O-B-9255 (F28-4Ko/19) strain SCPM-O-B-9255 (F28-4Ko/19) | WGS | PRJNA269675 | SAMN18437207 | 5947522 | 55.75 |
| 1134687.293 | *Klebsiella michiganensis* strain SCPM-O-B-9251 (F12-4Ko/19) strain SCPM-O-B-9251 (F12-4Ko/19) strain SCPM-O-B-9251 (F12-4Ko/19) | WGS | PRJNA269675 | SAMN18437203 | 5942632 | 55.76 |
| 1134687.294 | *Klebsiella michiganensis* strain SCPM-O-B-9252 (Z12Ko/19) strain SCPM-O-B-9252 (Z12Ko/19) strain SCPM-O-B-9252 (Z12Ko/19) | WGS | PRJNA269675 | SAMN18437204 | 5985423 | 55.65 |
| 1134687.296 | *Klebsiella michiganensis* strain CZ598 | Complete | PRJNA722384 | SAMN18753391 | 6494581 | 55.41 |
| 1134687.297 | *Klebsiella michiganensis* strain JGM22 | WGS | PRJNA687492 | SAMN17146120 | 5847855 | 55.57 |
| 1134687.299 | *Klebsiella michiganensis* strain Survcare371 | WGS | PRJNA692829 | SAMN17373049 | 6279656 | 55.72 |
| 1134687.300 | *Klebsiella michiganensis* strain Survcare365 | WGS | PRJNA692829 | SAMN17373043 | 6250448 | 55.63 |
| 1134687.301 | *Klebsiella michiganensis* strain NRZ-41473 | WGS | PRJNA692829 | SAMN17371848 | 6024865 | 55.99 |
| 1134687.302 | *Klebsiella michiganensis* strain NRZ-36521 | WGS | PRJNA692829 | SAMN17371824 | 5968477 | 55.93 |
| 1134687.303 | *Klebsiella michiganensis* strain NRZ-36825 | WGS | PRJNA692829 | SAMN17371826 | 5974612 | 55.94 |
| 1134687.304 | *Klebsiella michiganensis* strain NRZ-36826 | WGS | PRJNA692829 | SAMN17371827 | 5982510 | 55.93 |
| 1134687.305 | *Klebsiella michiganensis* strain NRZ-36109 | WGS | PRJNA692829 | SAMN17371821 | 6067990 | 55.99 |
| 1134687.306 | *Klebsiella michiganensis* strain NRZ-36455 | WGS | PRJNA692829 | SAMN17371823 | 6074451 | 56.00 |
| 1134687.307 | *Klebsiella michiganensis* strain Survcare276 | WGS | PRJNA692829 | SAMN17372164 | 6171347 | 55.98 |
| 1134687.308 | *Klebsiella michiganensis* strain Survcare126 | WGS | PRJNA692829 | SAMN17372045 | 5970061 | 55.94 |
| 1134687.309 | *Klebsiella michiganensis* strain Survcare124 | WGS | PRJNA692829 | SAMN17372043 | 5965845 | 55.93 |
| 1134687.310 | *Klebsiella michiganensis* strain SurvCare087 | WGS | PRJNA692829 | SAMN17372009 | 5962521 | 55.94 |
| 1134687.315 | *Klebsiella michiganensis* strain JNQH491 | Complete | PRJNA702614 | SAMN19186367 | 6552775 | 55.39 |
| 1134687.318 | *Klebsiella michiganensis* strain xz176 | WGS | PRJNA739673 | SAMN19797155 | 6176425 | 55.84 |
| 1134687.320 | *Klebsiella michiganensis* strain Colony483 | Complete | PRJNA668870 | SAMN17774997 | 6350720 | 56.49 |
| 1134687.321 | *Klebsiella michiganensis* strain Colony351 | Complete | PRJNA668870 | SAMN17774995 | 6337738 | 56.62 |
| 1134687.322 | *Klebsiella michiganensis* strain Colony517 | Complete | PRJNA668870 | SAMN17774996 | 6344682 | 56.63 |
| 1134687.323 | *Klebsiella michiganensis* strain 2513.3 strain CVUAS 2513.3 | WGS | PRJNA392781 | SAMN07310302 | 6083282 | 55.83 |
| 1134687.326 | *Klebsiella michiganensis* strain 2404.3 strain CVUAS 2404.3 | WGS | PRJNA392783 | SAMN07310307 | 5919331 | 55.89 |
| 1134687.327 | *Klebsiella michiganensis* strain 6675.2 strain CVUAS 6675.2 | WGS | PRJNA393157 | SAMN07319196 | 5982572 | 55.98 |
| 1134687.328 | *Klebsiella michiganensis* strain 9732.2 strain CVUAS 9732.2 | WGS | PRJNA393153 | SAMN07319192 | 6230074 | 55.93 |
| 1134687.331 | *Klebsiella michiganensis* strain K210011 | WGS | PRJNA738449 | SAMN19728927 | 6936928 | 54.83 |
| 1134687.332 | *Klebsiella michiganensis* strain CVUAS 11487 | WGS | PRJNA420635 | SAMN08117042 | 5791538 | 56.20 |
| 1134687.333 | *Klebsiella michiganensis* strain CVUAS 11699 | WGS | PRJNA431092 | SAMN08383930 | 6100159 | 55.75 |
| 1134687.334 | *Klebsiella michiganensis* strain CVUAS 11486 | WGS | PRJNA420636 | SAMN08117040 | 5975234 | 55.75 |
| 1134687.335 | *Klebsiella michiganensis* strain CVUAS 11390 | WGS | PRJNA420640 | SAMN08116998 | 6266365 | 55.67 |
| 1134687.336 | *Klebsiella michiganensis* strain CVUAS 11560.2 | WGS | PRJNA477562 | SAMN09475183 | 5947013 | 56.08 |
| 1134687.337 | *Klebsiella michiganensis* strain Y18 | WGS | PRJNA612981 | SAMN14389612 | 5961812 | 55.97 |
| 1134687.338 | *Klebsiella michiganensis* strain Y16 | WGS | PRJNA612981 | SAMN14389610 | 5961144 | 55.97 |
| 1134687.339 | *Klebsiella michiganensis* strain Y14 | WGS | PRJNA612981 | SAMN14389608 | 5962494 | 55.97 |
| 1134687.340 | *Klebsiella michiganensis* strain Y01 | WGS | PRJNA612981 | SAMN14389595 | 6253821 | 55.62 |
| 1134687.341 | *Klebsiella michiganensis* strain KE4018 | WGS | PRJNA546126 | SAMN12349705 | 6266630 | 55.70 |
| 1134687.342 | *Klebsiella michiganensis* strain CCRI-24235 | Complete | PRJNA744893 | SAMN20153793 | 6128315 | 55.92 |
| 1134687.343 | *Klebsiella michiganensis* strain 141125-16 | WGS | PRJNA543274 | SAMN12212121 | 5684378 | 56.42 |
| 1134687.344 | *Klebsiella michiganensis* strain DSM 103279 | WGS | PRJNA543274 | SAMN12212321 | 5717307 | 56.09 |
| 1134687.345 | *Klebsiella michiganensis* strain 800126-16 | WGS | PRJNA543274 | SAMN12212298 | 6282348 | 55.92 |
| 1134687.346 | *Klebsiella michiganensis* strain 720807-16 | WGS | PRJNA543274 | SAMN12212295 | 5924361 | 56.19 |
| 1134687.347 | *Klebsiella michiganensis* strain 710217-17 | WGS | PRJNA543274 | SAMN12212280 | 6071618 | 56.02 |
| 1134687.348 | *Klebsiella michiganensis* strain 707154-17 | WGS | PRJNA543274 | SAMN12212271 | 6389627 | 55.57 |
| 1134687.349 | *Klebsiella michiganensis* strain 708990-17 | WGS | PRJNA543274 | SAMN12212274 | 5791981 | 56.33 |
| 1134687.350 | *Klebsiella michiganensis* strain 707021-17 | WGS | PRJNA543274 | SAMN12212270 | 6521349 | 55.73 |
| 1134687.351 | *Klebsiella michiganensis* strain 706508-16 | WGS | PRJNA543274 | SAMN12212269 | 6101954 | 55.97 |
| 1134687.352 | *Klebsiella michiganensis* strain 705849-17 | WGS | PRJNA543274 | SAMN12212267 | 5836689 | 56.15 |
| 1134687.353 | *Klebsiella michiganensis* strain 705823-17 | WGS | PRJNA543274 | SAMN12212266 | 5691638 | 56.15 |
| 1134687.354 | *Klebsiella michiganensis* strain 620286643710 | WGS | PRJNA543274 | SAMN12212090 | 5873832 | 56.21 |
| 1134687.355 | *Klebsiella michiganensis* strain 620288768518 | WGS | PRJNA543274 | SAMN12212091 | 5787403 | 55.84 |
| 1134687.356 | *Klebsiella michiganensis* strain 620285505921 | WGS | PRJNA543274 | SAMN12212088 | 6461583 | 55.58 |
| 1134687.357 | *Klebsiella michiganensis* strain 620278998915 | WGS | PRJNA543274 | SAMN12212086 | 6147693 | 55.85 |
| 1134687.359 | *Klebsiella michiganensis* strain 620277132808 | WGS | PRJNA543274 | SAMN12212085 | 6134053 | 55.95 |
| 1134687.360 | *Klebsiella michiganensis* strain 620267602911 | WGS | PRJNA543274 | SAMN12212083 | 6108085 | 55.99 |
| 1134687.361 | *Klebsiella michiganensis* strain 620272244309 | WGS | PRJNA543274 | SAMN12212084 | 6146099 | 55.93 |
| 1134687.362 | *Klebsiella michiganensis* strain 620267298408 | WGS | PRJNA543274 | SAMN12212082 | 6042413 | 55.98 |
| 1134687.363 | *Klebsiella michiganensis* strain 620263865211 | WGS | PRJNA543274 | SAMN12212080 | 6231065 | 55.90 |
| 1134687.364 | *Klebsiella michiganensis* strain 620264432508 | WGS | PRJNA543274 | SAMN12212081 | 6224406 | 55.92 |
| 1134687.365 | *Klebsiella michiganensis* strain 620262583611 | WGS | PRJNA543274 | SAMN12212077 | 6661763 | 55.66 |
| 1134687.366 | *Klebsiella michiganensis* strain 620262927814 | WGS | PRJNA543274 | SAMN12212078 | 5899860 | 55.75 |
| 1134687.367 | *Klebsiella michiganensis* strain 620262455609 | WGS | PRJNA543274 | SAMN12212076 | 5802032 | 56.18 |
| 1134687.368 | *Klebsiella michiganensis* strain 620260277613 | WGS | PRJNA543274 | SAMN12212073 | 6069439 | 56.01 |
| 1134687.369 | *Klebsiella michiganensis* strain 620256896681 | WGS | PRJNA543274 | SAMN12212070 | 5983308 | 56.04 |
| 1134687.370 | *Klebsiella michiganensis* strain 620203084113 | WGS | PRJNA543274 | SAMN12212067 | 6052798 | 55.95 |
| 1134687.371 | *Klebsiella michiganensis* strain 612478-16 | WGS | PRJNA543274 | SAMN12212240 | 6217654 | 55.89 |
| 1134687.372 | *Klebsiella michiganensis* strain 608288-17 | WGS | PRJNA543274 | SAMN12212231 | 5901917 | 56.29 |
| 1134687.373 | *Klebsiella michiganensis* strain 607815-17 | WGS | PRJNA543274 | SAMN12212227 | 5993870 | 56.04 |
| 1134687.374 | *Klebsiella michiganensis* strain 605924-17 | WGS | PRJNA543274 | SAMN12212224 | 5942114 | 56.03 |
| 1134687.375 | *Klebsiella michiganensis* strain 603572-17 | WGS | PRJNA543274 | SAMN12212221 | 5967510 | 56.16 |
| 1134687.376 | *Klebsiella michiganensis* strain 602734-17 | WGS | PRJNA543274 | SAMN12212220 | 5981490 | 55.94 |
| 1134687.377 | *Klebsiella michiganensis* strain 505106-17 | WGS | PRJNA543274 | SAMN12212218 | 5770399 | 56.26 |
| 1134687.378 | *Klebsiella michiganensis* strain 502761-17 | WGS | PRJNA543274 | SAMN12212213 | 6071740 | 55.98 |
| 1134687.379 | *Klebsiella michiganensis* strain 502790-17 | WGS | PRJNA543274 | SAMN12212214 | 6142124 | 55.94 |
| 1134687.380 | *Klebsiella michiganensis* strain 502565-17 | WGS | PRJNA543274 | SAMN12212209 | 6025297 | 55.96 |
| 1134687.381 | *Klebsiella michiganensis* strain 501671-17 | WGS | PRJNA543274 | SAMN12212207 | 6119855 | 55.90 |
| 1134687.382 | *Klebsiella michiganensis* strain 500137-17 | WGS | PRJNA543274 | SAMN12212205 | 6202585 | 55.88 |
| 1134687.383 | *Klebsiella michiganensis* strain 44-2360-1 | WGS | PRJNA543274 | SAMN12212203 | 6192538 | 55.85 |
| 1134687.384 | *Klebsiella michiganensis* strain 401153-17 | WGS | PRJNA543274 | SAMN12212157 | 6523653 | 55.75 |
| 1134687.385 | *Klebsiella michiganensis* strain 400041-17 | WGS | PRJNA543274 | SAMN12212136 | 5951617 | 56.16 |
| 1134687.386 | *Klebsiella michiganensis* strain 170070391-17 | WGS | PRJNA543274 | SAMN12212123 | 6022597 | 56.00 |
| 1134687.387 | *Klebsiella michiganensis* strain 128489-17 | WGS | PRJNA543274 | SAMN12212107 | 6029859 | 56.14 |
| 1134687.388 | *Klebsiella michiganensis* strain 126192-17 | WGS | PRJNA543274 | SAMN12212105 | 5928541 | 56.11 |
| 1134687.389 | *Klebsiella michiganensis* strain 111734-17 | WGS | PRJNA543274 | SAMN12212099 | 5949285 | 55.90 |
| 1134687.390 | *Klebsiella michiganensis* strain 109680-17 | WGS | PRJNA543274 | SAMN12212095 | 6194579 | 55.95 |
| 1134687.392 | *Klebsiella michiganensis* strain Y18 | WGS | PRJNA612981 | SAMN14389612 | 5961812 | 55.97 |
| 1134687.393 | *Klebsiella michiganensis* strain Y16 | WGS | PRJNA612981 | SAMN14389610 | 5961144 | 55.97 |
| 1134687.394 | *Klebsiella michiganensis* strain Y14 | WGS | PRJNA612981 | SAMN14389608 | 5962494 | 55.97 |
| 1134687.395 | *Klebsiella michiganensis* strain Y01 | WGS | PRJNA612981 | SAMN14389595 | 6253821 | 55.62 |
| 1134687.396 | *Klebsiella michiganensis* strain KE4018 | WGS | PRJNA546126 | SAMN12349705 | 6266630 | 55.70 |
| 1134687.397 | *Klebsiella michiganensis* strain CCRI-24235 | Complete | PRJNA744893 | SAMN20153793 | 6128315 | 55.92 |
| 1134687.398 | *Klebsiella michiganensis* strain DSM 103279 | WGS | PRJNA543274 | SAMN12212321 | 5717307 | 56.09 |
| 1134687.399 | *Klebsiella michiganensis* strain 141125-16 | WGS | PRJNA543274 | SAMN12212121 | 5684378 | 56.42 |
| 1134687.400 | *Klebsiella michiganensis* strain 800126-16 | WGS | PRJNA543274 | SAMN12212298 | 6282348 | 55.92 |
| 1134687.401 | *Klebsiella michiganensis* strain 720807-16 | WGS | PRJNA543274 | SAMN12212295 | 5924361 | 56.19 |
| 1134687.402 | *Klebsiella michiganensis* strain 710217-17 | WGS | PRJNA543274 | SAMN12212280 | 6071618 | 56.02 |
| 1134687.403 | *Klebsiella michiganensis* strain 708990-17 | WGS | PRJNA543274 | SAMN12212274 | 5791981 | 56.33 |
| 1134687.404 | *Klebsiella michiganensis* strain 707154-17 | WGS | PRJNA543274 | SAMN12212271 | 6389627 | 55.57 |
| 1134687.405 | *Klebsiella michiganensis* strain 707021-17 | WGS | PRJNA543274 | SAMN12212270 | 6521349 | 55.73 |
| 1134687.406 | *Klebsiella michiganensis* strain 706508-16 | WGS | PRJNA543274 | SAMN12212269 | 6101954 | 55.97 |
| 1134687.407 | *Klebsiella michiganensis* strain 705849-17 | WGS | PRJNA543274 | SAMN12212267 | 5836689 | 56.15 |
| 1134687.408 | *Klebsiella michiganensis* strain 705823-17 | WGS | PRJNA543274 | SAMN12212266 | 5691638 | 56.15 |
| 1134687.409 | *Klebsiella michiganensis* strain 620286643710 | WGS | PRJNA543274 | SAMN12212090 | 5873832 | 56.21 |
| 1134687.410 | *Klebsiella michiganensis* strain 620288768518 | WGS | PRJNA543274 | SAMN12212091 | 5787403 | 55.84 |
| 1134687.411 | *Klebsiella michiganensis* strain 620285505921 | WGS | PRJNA543274 | SAMN12212088 | 6461583 | 55.58 |
| 1134687.412 | *Klebsiella michiganensis* strain 620278998915 | WGS | PRJNA543274 | SAMN12212086 | 6147693 | 55.85 |
| 1134687.414 | *Klebsiella michiganensis* strain 620277132808 | WGS | PRJNA543274 | SAMN12212085 | 6134053 | 55.95 |
| 1134687.415 | *Klebsiella michiganensis* strain 620267298408 | WGS | PRJNA543274 | SAMN12212082 | 6042413 | 55.98 |
| 1134687.416 | *Klebsiella michiganensis* strain 620272244309 | WGS | PRJNA543274 | SAMN12212084 | 6146099 | 55.93 |
| 1134687.417 | *Klebsiella michiganensis* strain 620267602911 | WGS | PRJNA543274 | SAMN12212083 | 6108085 | 55.99 |
| 1134687.418 | *Klebsiella michiganensis* strain 620263865211 | WGS | PRJNA543274 | SAMN12212080 | 6231065 | 55.90 |
| 1134687.419 | *Klebsiella michiganensis* strain 620264432508 | WGS | PRJNA543274 | SAMN12212081 | 6224406 | 55.92 |
| 1134687.420 | *Klebsiella michiganensis* strain 620262927814 | WGS | PRJNA543274 | SAMN12212078 | 5899860 | 55.75 |
| 1134687.421 | *Klebsiella michiganensis* strain 620262455609 | WGS | PRJNA543274 | SAMN12212076 | 5802032 | 56.18 |
| 1134687.422 | *Klebsiella michiganensis* strain 620260277613 | WGS | PRJNA543274 | SAMN12212073 | 6069439 | 56.01 |
| 1134687.423 | *Klebsiella michiganensis* strain 620262583611 | WGS | PRJNA543274 | SAMN12212077 | 6661763 | 55.66 |
| 1134687.424 | *Klebsiella michiganensis* strain 620256896681 | WGS | PRJNA543274 | SAMN12212070 | 5983308 | 56.04 |
| 1134687.425 | *Klebsiella michiganensis* strain 620203084113 | WGS | PRJNA543274 | SAMN12212067 | 6052798 | 55.95 |
| 1134687.426 | *Klebsiella michiganensis* strain 612478-16 | WGS | PRJNA543274 | SAMN12212240 | 6217654 | 55.89 |
| 1134687.427 | *Klebsiella michiganensis* strain 608288-17 | WGS | PRJNA543274 | SAMN12212231 | 5901917 | 56.29 |
| 1134687.428 | *Klebsiella michiganensis* strain 605924-17 | WGS | PRJNA543274 | SAMN12212224 | 5942114 | 56.03 |
| 1134687.429 | *Klebsiella michiganensis* strain 607815-17 | WGS | PRJNA543274 | SAMN12212227 | 5993870 | 56.04 |
| 1134687.430 | *Klebsiella michiganensis* strain 603572-17 | WGS | PRJNA543274 | SAMN12212221 | 5967510 | 56.16 |
| 1134687.431 | *Klebsiella michiganensis* strain 602734-17 | WGS | PRJNA543274 | SAMN12212220 | 5981490 | 55.94 |
| 1134687.432 | *Klebsiella michiganensis* strain 505106-17 | WGS | PRJNA543274 | SAMN12212218 | 5770399 | 56.26 |
| 1134687.433 | *Klebsiella michiganensis* strain 502790-17 | WGS | PRJNA543274 | SAMN12212214 | 6142124 | 55.94 |
| 1134687.434 | *Klebsiella michiganensis* strain 502761-17 | WGS | PRJNA543274 | SAMN12212213 | 6071740 | 55.98 |
| 1134687.435 | *Klebsiella michiganensis* strain 502565-17 | WGS | PRJNA543274 | SAMN12212209 | 6025297 | 55.96 |
| 1134687.436 | *Klebsiella michiganensis* strain 501671-17 | WGS | PRJNA543274 | SAMN12212207 | 6119855 | 55.90 |
| 1134687.437 | *Klebsiella michiganensis* strain 500137-17 | WGS | PRJNA543274 | SAMN12212205 | 6202585 | 55.88 |
| 1134687.438 | *Klebsiella michiganensis* strain 44-2360-1 | WGS | PRJNA543274 | SAMN12212203 | 6192538 | 55.85 |
| 1134687.439 | *Klebsiella michiganensis* strain 401153-17 | WGS | PRJNA543274 | SAMN12212157 | 6523653 | 55.75 |
| 1134687.440 | *Klebsiella michiganensis* strain 400041-17 | WGS | PRJNA543274 | SAMN12212136 | 5951617 | 56.16 |
| 1134687.441 | *Klebsiella michiganensis* strain 128489-17 | WGS | PRJNA543274 | SAMN12212107 | 6029859 | 56.14 |
| 1134687.442 | *Klebsiella michiganensis* strain 170070391-17 | WGS | PRJNA543274 | SAMN12212123 | 6022597 | 56.00 |
| 1134687.443 | *Klebsiella michiganensis* strain 126192-17 | WGS | PRJNA543274 | SAMN12212105 | 5928541 | 56.11 |
| 1134687.444 | *Klebsiella michiganensis* strain 111734-17 | WGS | PRJNA543274 | SAMN12212099 | 5949285 | 55.90 |
| 1134687.445 | *Klebsiella michiganensis* strain 109680-17 | WGS | PRJNA543274 | SAMN12212095 | 6194579 | 55.95 |
| 1134687.446 | *Klebsiella michiganensis* strain BEITU-N172 | WGS | PRJNA612328 | SAMN15754140 | 6127465 | 55.82 |
| 1134687.447 | *Klebsiella michiganensis* strain Y18 | WGS | PRJNA612981 | SAMN14389612 | 5961812 | 55.97 |
| 1134687.448 | *Klebsiella michiganensis* strain Y16 | WGS | PRJNA612981 | SAMN14389610 | 5961144 | 55.97 |
| 1134687.449 | *Klebsiella michiganensis* strain Y14 | WGS | PRJNA612981 | SAMN14389608 | 5962494 | 55.97 |
| 1134687.450 | *Klebsiella michiganensis* strain Y01 | WGS | PRJNA612981 | SAMN14389595 | 6253821 | 55.62 |
| 1134687.451 | *Klebsiella michiganensis* strain KE4018 | WGS | PRJNA546126 | SAMN12349705 | 6266630 | 55.70 |
| 1134687.452 | *Klebsiella michiganensis* strain CCRI-24235 | Complete | PRJNA744893 | SAMN20153793 | 6128315 | 55.92 |
| 1134687.453 | *Klebsiella michiganensis* strain 141125-16 | WGS | PRJNA543274 | SAMN12212121 | 5684378 | 56.42 |
| 1134687.454 | *Klebsiella michiganensis* strain DSM 103279 | WGS | PRJNA543274 | SAMN12212321 | 5717307 | 56.09 |
| 1134687.455 | *Klebsiella michiganensis* strain 800126-16 | WGS | PRJNA543274 | SAMN12212298 | 6282348 | 55.92 |
| 1134687.456 | *Klebsiella michiganensis* strain 720807-16 | WGS | PRJNA543274 | SAMN12212295 | 5924361 | 56.19 |
| 1134687.457 | *Klebsiella michiganensis* strain 710217-17 | WGS | PRJNA543274 | SAMN12212280 | 6071618 | 56.02 |
| 1134687.458 | *Klebsiella michiganensis* strain 707154-17 | WGS | PRJNA543274 | SAMN12212271 | 6389627 | 55.57 |
| 1134687.459 | *Klebsiella michiganensis* strain 708990-17 | WGS | PRJNA543274 | SAMN12212274 | 5791981 | 56.33 |
| 1134687.460 | *Klebsiella michiganensis* strain 707021-17 | WGS | PRJNA543274 | SAMN12212270 | 6521349 | 55.73 |
| 1134687.461 | *Klebsiella michiganensis* strain 706508-16 | WGS | PRJNA543274 | SAMN12212269 | 6101954 | 55.97 |
| 1134687.462 | *Klebsiella michiganensis* strain 705823-17 | WGS | PRJNA543274 | SAMN12212266 | 5691638 | 56.15 |
| 1134687.463 | *Klebsiella michiganensis* strain 705849-17 | WGS | PRJNA543274 | SAMN12212267 | 5836689 | 56.15 |
| 1134687.464 | *Klebsiella michiganensis* strain 620286643710 | WGS | PRJNA543274 | SAMN12212090 | 5873832 | 56.21 |
| 1134687.465 | *Klebsiella michiganensis* strain 620285505921 | WGS | PRJNA543274 | SAMN12212088 | 6461583 | 55.58 |
| 1134687.466 | *Klebsiella michiganensis* strain 620288768518 | WGS | PRJNA543274 | SAMN12212091 | 5787403 | 55.84 |
| 1134687.467 | *Klebsiella michiganensis* strain 620278998915 | WGS | PRJNA543274 | SAMN12212086 | 6147693 | 55.85 |
| 1134687.468 | *Klebsiella michiganensis* strain 620277132808 | WGS | PRJNA543274 | SAMN12212085 | 6134053 | 55.95 |
| 1134687.470 | *Klebsiella michiganensis* strain 620267602911 | WGS | PRJNA543274 | SAMN12212083 | 6108085 | 55.99 |
| 1134687.471 | *Klebsiella michiganensis* strain 620272244309 | WGS | PRJNA543274 | SAMN12212084 | 6146099 | 55.93 |
| 1134687.472 | *Klebsiella michiganensis* strain 620267298408 | WGS | PRJNA543274 | SAMN12212082 | 6042413 | 55.98 |
| 1134687.473 | *Klebsiella michiganensis* strain 620263865211 | WGS | PRJNA543274 | SAMN12212080 | 6231065 | 55.90 |
| 1134687.474 | *Klebsiella michiganensis* strain 620264432508 | WGS | PRJNA543274 | SAMN12212081 | 6224406 | 55.92 |
| 1134687.475 | *Klebsiella michiganensis* strain 620262583611 | WGS | PRJNA543274 | SAMN12212077 | 6661763 | 55.66 |
| 1134687.476 | *Klebsiella michiganensis* strain 620262455609 | WGS | PRJNA543274 | SAMN12212076 | 5802032 | 56.18 |
| 1134687.477 | *Klebsiella michiganensis* strain 620262927814 | WGS | PRJNA543274 | SAMN12212078 | 5899860 | 55.75 |
| 1134687.478 | *Klebsiella michiganensis* strain 620260277613 | WGS | PRJNA543274 | SAMN12212073 | 6069439 | 56.01 |
| 1134687.479 | *Klebsiella michiganensis* strain 620256896681 | WGS | PRJNA543274 | SAMN12212070 | 5983308 | 56.04 |
| 1134687.480 | *Klebsiella michiganensis* strain 620203084113 | WGS | PRJNA543274 | SAMN12212067 | 6052798 | 55.95 |
| 1134687.481 | *Klebsiella michiganensis* strain 612478-16 | WGS | PRJNA543274 | SAMN12212240 | 6217654 | 55.89 |
| 1134687.482 | *Klebsiella michiganensis* strain 608288-17 | WGS | PRJNA543274 | SAMN12212231 | 5901917 | 56.29 |
| 1134687.483 | *Klebsiella michiganensis* strain 607815-17 | WGS | PRJNA543274 | SAMN12212227 | 5993870 | 56.04 |
| 1134687.484 | *Klebsiella michiganensis* strain 605924-17 | WGS | PRJNA543274 | SAMN12212224 | 5942114 | 56.03 |
| 1134687.485 | *Klebsiella michiganensis* strain 603572-17 | WGS | PRJNA543274 | SAMN12212221 | 5967510 | 56.16 |
| 1134687.486 | *Klebsiella michiganensis* strain 602734-17 | WGS | PRJNA543274 | SAMN12212220 | 5981490 | 55.94 |
| 1134687.487 | *Klebsiella michiganensis* strain 505106-17 | WGS | PRJNA543274 | SAMN12212218 | 5770399 | 56.26 |
| 1134687.488 | *Klebsiella michiganensis* strain 502761-17 | WGS | PRJNA543274 | SAMN12212213 | 6071740 | 55.98 |
| 1134687.489 | *Klebsiella michiganensis* strain 502790-17 | WGS | PRJNA543274 | SAMN12212214 | 6142124 | 55.94 |
| 1134687.490 | *Klebsiella michiganensis* strain 502565-17 | WGS | PRJNA543274 | SAMN12212209 | 6025297 | 55.96 |
| 1134687.491 | *Klebsiella michiganensis* strain 501671-17 | WGS | PRJNA543274 | SAMN12212207 | 6119855 | 55.90 |
| 1134687.492 | *Klebsiella michiganensis* strain 500137-17 | WGS | PRJNA543274 | SAMN12212205 | 6202585 | 55.88 |
| 1134687.493 | *Klebsiella michiganensis* strain 44-2360-1 | WGS | PRJNA543274 | SAMN12212203 | 6192538 | 55.85 |
| 1134687.494 | *Klebsiella michiganensis* strain 401153-17 | WGS | PRJNA543274 | SAMN12212157 | 6523653 | 55.75 |
| 1134687.495 | *Klebsiella michiganensis* strain 400041-17 | WGS | PRJNA543274 | SAMN12212136 | 5951617 | 56.16 |
| 1134687.496 | *Klebsiella michiganensis* strain 170070391-17 | WGS | PRJNA543274 | SAMN12212123 | 6022597 | 56.00 |
| 1134687.497 | *Klebsiella michiganensis* strain 128489-17 | WGS | PRJNA543274 | SAMN12212107 | 6029859 | 56.14 |
| 1134687.498 | *Klebsiella michiganensis* strain 126192-17 | WGS | PRJNA543274 | SAMN12212105 | 5928541 | 56.11 |
| 1134687.499 | *Klebsiella michiganensis* strain 111734-17 | WGS | PRJNA543274 | SAMN12212099 | 5949285 | 55.90 |
| 1134687.500 | *Klebsiella michiganensis* strain 109680-17 | WGS | PRJNA543274 | SAMN12212095 | 6194579 | 55.95 |
| 1134687.501 | *Klebsiella michiganensis* strain BEITU-N172 | WGS | PRJNA612328 | SAMN15754140 | 6127465 | 55.82 |
| 1134687.502 | *Klebsiella michiganensis* strain 53828CZ | Complete | PRJNA772913 | SAMN22377421 | 6455282 | 55.61 |
| 1134687.507 | *Klebsiella michiganensis* strain Y18 | WGS | PRJNA612981 | SAMN14389612 | 5961812 | 55.97 |
| 1134687.508 | *Klebsiella michiganensis* strain Y16 | WGS | PRJNA612981 | SAMN14389610 | 5961144 | 55.97 |
| 1134687.509 | *Klebsiella michiganensis* strain Y14 | WGS | PRJNA612981 | SAMN14389608 | 5962494 | 55.97 |
| 1134687.510 | *Klebsiella michiganensis* strain Y01 | WGS | PRJNA612981 | SAMN14389595 | 6253821 | 55.62 |
| 1134687.511 | *Klebsiella michiganensis* strain KE4018 | WGS | PRJNA546126 | SAMN12349705 | 6266630 | 55.70 |
| 1134687.512 | *Klebsiella michiganensis* strain CCRI-24235 | Complete | PRJNA744893 | SAMN20153793 | 6128315 | 55.92 |
| 1134687.513 | *Klebsiella michiganensis* strain 141125-16 | WGS | PRJNA543274 | SAMN12212121 | 5684378 | 56.42 |
| 1134687.514 | *Klebsiella michiganensis* strain DSM 103279 | WGS | PRJNA543274 | SAMN12212321 | 5717307 | 56.09 |
| 1134687.515 | *Klebsiella michiganensis* strain 800126-16 | WGS | PRJNA543274 | SAMN12212298 | 6282348 | 55.92 |
| 1134687.516 | *Klebsiella michiganensis* strain 720807-16 | WGS | PRJNA543274 | SAMN12212295 | 5924361 | 56.19 |
| 1134687.517 | *Klebsiella michiganensis* strain 710217-17 | WGS | PRJNA543274 | SAMN12212280 | 6071618 | 56.02 |
| 1134687.518 | *Klebsiella michiganensis* strain 707154-17 | WGS | PRJNA543274 | SAMN12212271 | 6389627 | 55.57 |
| 1134687.519 | *Klebsiella michiganensis* strain 708990-17 | WGS | PRJNA543274 | SAMN12212274 | 5791981 | 56.33 |
| 1134687.520 | *Klebsiella michiganensis* strain 706508-16 | WGS | PRJNA543274 | SAMN12212269 | 6101954 | 55.97 |
| 1134687.521 | *Klebsiella michiganensis* strain 707021-17 | WGS | PRJNA543274 | SAMN12212270 | 6521349 | 55.73 |
| 1134687.522 | *Klebsiella michiganensis* strain 705849-17 | WGS | PRJNA543274 | SAMN12212267 | 5836689 | 56.15 |
| 1134687.523 | *Klebsiella michiganensis* strain 705823-17 | WGS | PRJNA543274 | SAMN12212266 | 5691638 | 56.15 |
| 1134687.524 | *Klebsiella michiganensis* strain 620286643710 | WGS | PRJNA543274 | SAMN12212090 | 5873832 | 56.21 |
| 1134687.525 | *Klebsiella michiganensis* strain 620288768518 | WGS | PRJNA543274 | SAMN12212091 | 5787403 | 55.84 |
| 1134687.526 | *Klebsiella michiganensis* strain 620285505921 | WGS | PRJNA543274 | SAMN12212088 | 6461583 | 55.58 |
| 1134687.527 | *Klebsiella michiganensis* strain 620278998915 | WGS | PRJNA543274 | SAMN12212086 | 6147693 | 55.85 |
| 1134687.529 | *Klebsiella michiganensis* strain 620277132808 | WGS | PRJNA543274 | SAMN12212085 | 6134053 | 55.95 |
| 1134687.530 | *Klebsiella michiganensis* strain 620267602911 | WGS | PRJNA543274 | SAMN12212083 | 6108085 | 55.99 |
| 1134687.531 | *Klebsiella michiganensis* strain 620272244309 | WGS | PRJNA543274 | SAMN12212084 | 6146099 | 55.93 |
| 1134687.532 | *Klebsiella michiganensis* strain 620267298408 | WGS | PRJNA543274 | SAMN12212082 | 6042413 | 55.98 |
| 1134687.533 | *Klebsiella michiganensis* strain 620263865211 | WGS | PRJNA543274 | SAMN12212080 | 6231065 | 55.90 |
| 1134687.534 | *Klebsiella michiganensis* strain 620264432508 | WGS | PRJNA543274 | SAMN12212081 | 6224406 | 55.92 |
| 1134687.535 | *Klebsiella michiganensis* strain 620262583611 | WGS | PRJNA543274 | SAMN12212077 | 6661763 | 55.66 |
| 1134687.536 | *Klebsiella michiganensis* strain 620262927814 | WGS | PRJNA543274 | SAMN12212078 | 5899860 | 55.75 |
| 1134687.537 | *Klebsiella michiganensis* strain 620262455609 | WGS | PRJNA543274 | SAMN12212076 | 5802032 | 56.18 |
| 1134687.538 | *Klebsiella michiganensis* strain 620260277613 | WGS | PRJNA543274 | SAMN12212073 | 6069439 | 56.01 |
| 1134687.539 | *Klebsiella michiganensis* strain 620256896681 | WGS | PRJNA543274 | SAMN12212070 | 5983308 | 56.04 |
| 1134687.540 | *Klebsiella michiganensis* strain 620203084113 | WGS | PRJNA543274 | SAMN12212067 | 6052798 | 55.95 |
| 1134687.541 | *Klebsiella michiganensis* strain 612478-16 | WGS | PRJNA543274 | SAMN12212240 | 6217654 | 55.89 |
| 1134687.542 | *Klebsiella michiganensis* strain 608288-17 | WGS | PRJNA543274 | SAMN12212231 | 5901917 | 56.29 |
| 1134687.543 | *Klebsiella michiganensis* strain 607815-17 | WGS | PRJNA543274 | SAMN12212227 | 5993870 | 56.04 |
| 1134687.544 | *Klebsiella michiganensis* strain 605924-17 | WGS | PRJNA543274 | SAMN12212224 | 5942114 | 56.03 |
| 1134687.545 | *Klebsiella michiganensis* strain 603572-17 | WGS | PRJNA543274 | SAMN12212221 | 5967510 | 56.16 |
| 1134687.546 | *Klebsiella michiganensis* strain 602734-17 | WGS | PRJNA543274 | SAMN12212220 | 5981490 | 55.94 |
| 1134687.547 | *Klebsiella michiganensis* strain 505106-17 | WGS | PRJNA543274 | SAMN12212218 | 5770399 | 56.26 |
| 1134687.549 | *Klebsiella michiganensis* strain 502790-17 | WGS | PRJNA543274 | SAMN12212214 | 6142124 | 55.94 |
| 1134687.550 | *Klebsiella michiganensis* strain 502565-17 | WGS | PRJNA543274 | SAMN12212209 | 6025297 | 55.96 |
| 1134687.551 | *Klebsiella michiganensis* strain 501671-17 | WGS | PRJNA543274 | SAMN12212207 | 6119855 | 55.90 |
| 1134687.552 | *Klebsiella michiganensis* strain 500137-17 | WGS | PRJNA543274 | SAMN12212205 | 6202585 | 55.88 |
| 1134687.553 | *Klebsiella michiganensis* strain 44-2360-1 | WGS | PRJNA543274 | SAMN12212203 | 6192538 | 55.85 |
| 1134687.554 | *Klebsiella michiganensis* strain 401153-17 | WGS | PRJNA543274 | SAMN12212157 | 6523653 | 55.75 |
| 1134687.555 | *Klebsiella michiganensis* strain 400041-17 | WGS | PRJNA543274 | SAMN12212136 | 5951617 | 56.16 |
| 1134687.556 | *Klebsiella michiganensis* strain 170070391-17 | WGS | PRJNA543274 | SAMN12212123 | 6022597 | 56.00 |
| 1134687.557 | *Klebsiella michiganensis* strain 128489-17 | WGS | PRJNA543274 | SAMN12212107 | 6029859 | 56.14 |
| 1134687.558 | *Klebsiella michiganensis* strain 126192-17 | WGS | PRJNA543274 | SAMN12212105 | 5928541 | 56.11 |
| 1134687.559 | *Klebsiella michiganensis* strain 111734-17 | WGS | PRJNA543274 | SAMN12212099 | 5949285 | 55.90 |
| 1134687.560 | *Klebsiella michiganensis* strain 109680-17 | WGS | PRJNA543274 | SAMN12212095 | 6194579 | 55.95 |
| 1134687.561 | *Klebsiella michiganensis* strain BEITU-N172 | WGS | PRJNA612328 | SAMN15754140 | 6127465 | 55.82 |
| 1134687.562 | *Klebsiella michiganensis* strain 53828CZ | Complete | PRJNA772913 | SAMN22377421 | 6455282 | 55.61 |
| 1134687.563 | *Klebsiella michiganensis* strain CFS0697 | WGS | PRJNA786472 | SAMN23673799 | 6461268 | 55.23 |
| 1134687.569 | *Klebsiella michiganensis* MIN-086 | Complete | PRJNA769286 | SAMN22108523 | 6064047 | 55.74 |
| 1134687.570 | *Klebsiella michiganensis* Y18 | WGS | PRJNA612981 | SAMN14389612 | 5961812 | 55.97 |
| 1134687.571 | *Klebsiella michiganensis* Y16 | WGS | PRJNA612981 | SAMN14389610 | 5961144 | 55.97 |
| 1134687.572 | *Klebsiella michiganensis* Y14 | WGS | PRJNA612981 | SAMN14389608 | 5962494 | 55.97 |
| 1134687.573 | *Klebsiella michiganensis* Y01 | WGS | PRJNA612981 | SAMN14389595 | 6253821 | 55.62 |
| 1134687.574 | *Klebsiella michiganensis* KE4018 | WGS | PRJNA546126 | SAMN12349705 | 6266630 | 55.70 |
| 1134687.575 | *Klebsiella michiganensis* CCRI-24235 | Complete | PRJNA744893 | SAMN20153793 | 6128315 | 55.92 |
| 1134687.576 | *Klebsiella michiganensis* 53828CZ | Complete | PRJNA772913 | SAMN22377421 | 6455282 | 55.61 |
| 1134687.577 | *Klebsiella michiganensis* ARGID_31680 | WGS | PRJEB48990 | SAMEA11350720 | 6734851 | 55.17 |
| 1134687.578 | *Klebsiella michiganensis* ARGID_32776 | WGS | PRJEB48990 | SAMEA11350716 | 6126261 | 56.02 |
| 1134687.579 | *Klebsiella michiganensis* ARGID_31165 | WGS | PRJEB48990 | SAMEA11350711 | 6483293 | 55.74 |
| 1134687.580 | *Klebsiella michiganensis* 141125-16 | WGS | PRJNA543274 | SAMN12212121 | 5684378 | 56.42 |
| 1134687.581 | *Klebsiella michiganensis* DSM 103279 | WGS | PRJNA543274 | SAMN12212321 | 5717307 | 56.09 |
| 1134687.582 | *Klebsiella michiganensis* 800126-16 | WGS | PRJNA543274 | SAMN12212298 | 6282348 | 55.92 |
| 1134687.583 | *Klebsiella michiganensis* 720807-16 | WGS | PRJNA543274 | SAMN12212295 | 5924361 | 56.19 |
| 1134687.584 | *Klebsiella michiganensis* 710217-17 | WGS | PRJNA543274 | SAMN12212280 | 6071618 | 56.02 |
| 1134687.585 | *Klebsiella michiganensis* 707154-17 | WGS | PRJNA543274 | SAMN12212271 | 6389627 | 55.57 |
| 1134687.586 | *Klebsiella michiganensis* 708990-17 | WGS | PRJNA543274 | SAMN12212274 | 5791981 | 56.33 |
| 1134687.587 | *Klebsiella michiganensis* 707021-17 | WGS | PRJNA543274 | SAMN12212270 | 6521349 | 55.73 |
| 1134687.588 | *Klebsiella michiganensis* 706508-16 | WGS | PRJNA543274 | SAMN12212269 | 6101954 | 55.97 |
| 1134687.589 | *Klebsiella michiganensis* 705849-17 | WGS | PRJNA543274 | SAMN12212267 | 5836689 | 56.15 |
| 1134687.59 | *Klebsiella michiganensis* strain YD358 | WGS | PRJNA416908 | SAMN07974452 | 6663261 | 55.46 |
| 1134687.590 | *Klebsiella michiganensis* 705823-17 | WGS | PRJNA543274 | SAMN12212266 | 5691638 | 56.15 |
| 1134687.591 | *Klebsiella michiganensis* 620286643710 | WGS | PRJNA543274 | SAMN12212090 | 5873832 | 56.21 |
| 1134687.592 | *Klebsiella michiganensis* 620288768518 | WGS | PRJNA543274 | SAMN12212091 | 5787403 | 55.84 |
| 1134687.593 | *Klebsiella michiganensis* 620285505921 | WGS | PRJNA543274 | SAMN12212088 | 6461583 | 55.58 |
| 1134687.594 | *Klebsiella michiganensis* 620278998915 | WGS | PRJNA543274 | SAMN12212086 | 6147693 | 55.85 |
| 1134687.596 | *Klebsiella michiganensis* 620277132808 | WGS | PRJNA543274 | SAMN12212085 | 6134053 | 55.95 |
| 1134687.597 | *Klebsiella michiganensis* 620267602911 | WGS | PRJNA543274 | SAMN12212083 | 6108085 | 55.99 |
| 1134687.598 | *Klebsiella michiganensis* 620272244309 | WGS | PRJNA543274 | SAMN12212084 | 6146099 | 55.93 |
| 1134687.599 | *Klebsiella michiganensis* 620267298408 | WGS | PRJNA543274 | SAMN12212082 | 6042413 | 55.98 |
| 1134687.60 | *Klebsiella michiganensis* strain YDC736-2 | WGS | PRJNA416908 | SAMN07974482 | 6089209 | 55.98 |
| 1134687.600 | *Klebsiella michiganensis* 620263865211 | WGS | PRJNA543274 | SAMN12212080 | 6231065 | 55.90 |
| 1134687.601 | *Klebsiella michiganensis* 620264432508 | WGS | PRJNA543274 | SAMN12212081 | 6224406 | 55.92 |
| 1134687.602 | *Klebsiella michiganensis* 620262583611 | WGS | PRJNA543274 | SAMN12212077 | 6661763 | 55.66 |
| 1134687.603 | *Klebsiella michiganensis* 620262927814 | WGS | PRJNA543274 | SAMN12212078 | 5899860 | 55.75 |
| 1134687.604 | *Klebsiella michiganensis* 620262455609 | WGS | PRJNA543274 | SAMN12212076 | 5802032 | 56.18 |
| 1134687.605 | *Klebsiella michiganensis* 620260277613 | WGS | PRJNA543274 | SAMN12212073 | 6069439 | 56.01 |
| 1134687.606 | *Klebsiella michiganensis* 620256896681 | WGS | PRJNA543274 | SAMN12212070 | 5983308 | 56.04 |
| 1134687.607 | *Klebsiella michiganensis* 620203084113 | WGS | PRJNA543274 | SAMN12212067 | 6052798 | 55.95 |
| 1134687.608 | *Klebsiella michiganensis* 612478-16 | WGS | PRJNA543274 | SAMN12212240 | 6217654 | 55.89 |
| 1134687.609 | *Klebsiella michiganensis* 608288-17 | WGS | PRJNA543274 | SAMN12212231 | 5901917 | 56.29 |
| 1134687.61 | *Klebsiella michiganensis* strain DSM 25444 | WGS | PRJNA388837 | SAMN07187491 | 6193009 | 55.97 |
| 1134687.610 | *Klebsiella michiganensis* 607815-17 | WGS | PRJNA543274 | SAMN12212227 | 5993870 | 56.04 |
| 1134687.611 | *Klebsiella michiganensis* 605924-17 | WGS | PRJNA543274 | SAMN12212224 | 5942114 | 56.03 |
| 1134687.612 | *Klebsiella michiganensis* 603572-17 | WGS | PRJNA543274 | SAMN12212221 | 5967510 | 56.16 |
| 1134687.613 | *Klebsiella michiganensis* 602734-17 | WGS | PRJNA543274 | SAMN12212220 | 5981490 | 55.94 |
| 1134687.614 | *Klebsiella michiganensis* 505106-17 | WGS | PRJNA543274 | SAMN12212218 | 5770399 | 56.26 |
| 1134687.615 | *Klebsiella michiganensis* 502761-17 | WGS | PRJNA543274 | SAMN12212213 | 6071740 | 55.98 |
| 1134687.616 | *Klebsiella michiganensis* 502790-17 | WGS | PRJNA543274 | SAMN12212214 | 6142124 | 55.94 |
| 1134687.617 | *Klebsiella michiganensis* 502565-17 | WGS | PRJNA543274 | SAMN12212209 | 6025297 | 55.96 |
| 1134687.618 | *Klebsiella michiganensis* 501671-17 | WGS | PRJNA543274 | SAMN12212207 | 6119855 | 55.90 |
| 1134687.619 | *Klebsiella michiganensis* 500137-17 | WGS | PRJNA543274 | SAMN12212205 | 6202585 | 55.88 |
| 1134687.62 | *Klebsiella michiganensis* strain 2654 | WGS | PRJNA437380 | SAMN08661023 | 6049274 | 55.79 |
| 1134687.620 | *Klebsiella michiganensis* 44-2360-1 | WGS | PRJNA543274 | SAMN12212203 | 6192538 | 55.85 |
| 1134687.621 | *Klebsiella michiganensis* 401153-17 | WGS | PRJNA543274 | SAMN12212157 | 6523653 | 55.75 |
| 1134687.622 | *Klebsiella michiganensis* 400041-17 | WGS | PRJNA543274 | SAMN12212136 | 5951617 | 56.16 |
| 1134687.623 | *Klebsiella michiganensis* 170070391-17 | WGS | PRJNA543274 | SAMN12212123 | 6022597 | 56.00 |
| 1134687.624 | *Klebsiella michiganensis* 128489-17 | WGS | PRJNA543274 | SAMN12212107 | 6029859 | 56.14 |
| 1134687.625 | *Klebsiella michiganensis* 126192-17 | WGS | PRJNA543274 | SAMN12212105 | 5928541 | 56.11 |
| 1134687.626 | *Klebsiella michiganensis* 111734-17 | WGS | PRJNA543274 | SAMN12212099 | 5949285 | 55.90 |
| 1134687.627 | *Klebsiella michiganensis* 109680-17 | WGS | PRJNA543274 | SAMN12212095 | 6194579 | 55.95 |
| 1134687.628 | *Klebsiella michiganensis* CFS0697 | WGS | PRJNA786472 | SAMN23673799 | 6461268 | 55.23 |
| 1134687.629 | *Klebsiella michiganensis* Kox58 | Complete | PRJNA781656 | SAMN23286118 | 6355275 | 55.73 |
| 1134687.63 | *Klebsiella michiganensis* strain AR375 | Complete | PRJNA316321 | SAMN07291518 | 6525490 | 55.61 |
| 1134687.630 | *Klebsiella michiganensis* Kox101 | Complete | PRJNA781656 | SAMN23286126 | 6472862 | 55.77 |
| 1134687.631 | *Klebsiella michiganensis* K92 | Complete | PRJNA787404 | SAMN23799016 | 6108919 | 56.00 |
| 1134687.632 | *Klebsiella michiganensis* 8-1 | Complete | PRJNA787314 | SAMN23798052 | 6008113 | 56.07 |
| 1134687.633 | *Klebsiella michiganensis* BEITU-N172 | WGS | PRJNA612328 | SAMN15754140 | 6127465 | 55.82 |
| 1134687.634 | *Klebsiella michiganensis* KM41 | Complete | PRJNA791461 | SAMN24290086 | 6505209 | 55.19 |
| 1134687.635 | *Klebsiella michiganensis* A2 | WGS | PRJNA793885 | SAMN24592235 | 5896501 | 56.07 |
| 1134687.636 | *Klebsiella michiganensis* INSAq73 | WGS | PRJNA762299 | SAMN24244976 | 6253193 | 55.73 |
| 1134687.70 | *Klebsiella michiganensis* strain IS1015-72 | WGS | PRJNA451179 | SAMN08963301 | 6542284 | 55.81 |
| 1134687.71 | *Klebsiella michiganensis* strain XP-B1 | WGS | PRJNA289043 | SAMN03840808 | 6555694 | 55.65 |
| 1134687.72 | *Klebsiella michiganensis* strain 1016942 | WGS | PRJNA497260 | SAMN10252243 | 6597912 | 55.75 |
| 1134687.75 | *Klebsiella michiganensis* strain KMISG1 | WGS | PRJNA485881 | SAMN09829938 | 6107111 | 56.07 |
| 1134687.76 | *Klebsiella michiganensis* strain GEO_33_Up_A | WGS | PRJNA472583 | SAMN09289747 | 6847574 | 55.26 |
| 1134687.77 | *Klebsiella michiganensis* strain GEO_49_Down_A | WGS | PRJNA472583 | SAMN09289758 | 6394816 | 55.34 |
| 1134687.8 | *Klebsiella michiganensis* strain 97_38 | WGS | PRJNA353361 | SAMN06014568 | 6514001 | 55.65 |
| 1134687.9 | *Klebsiella michiganensis* strain CAV1755 | WGS | PRJNA246471 | SAMN03733822 | 7148482 | 55.46 |
| 1191061.3 | *Klebsiella oxytoca* E718 | Complete | PRJNA167370 | SAMN02603642 | 6450897 | 55.53 |
| 1308980.3 | *Klebsiella oxytoca* HKOPL1 | Complete | PRJNA194061 | SAMN03081460 | 5914407 | 55.90 |
| 1416754.3 | *Klebsiella oxytoca* H1g | WGS | PRJNA226202 | SAMN02729846 | 5840921 | 56.00 |
